# Ambient-Pressure Multischeme Chemical Ionization for Pesticide Detection: A MION-Orbitrap Mass Spectrometry Study

**DOI:** 10.1021/acsomega.4c11287

**Published:** 2025-05-23

**Authors:** Fariba Partovi, Joona Mikkilä, Siddharth Iyer, Jyri Mikkilä, Jussi Kontro, Suvi Ojanperä, Aleksei Shcherbinin, Matti Rissanen

**Affiliations:** † Karsa Ltd., A. I. Virtasen aukio 1, 00560 Helsinki, Finland; ‡ Aerosol Physics Laboratory, Physics Unit, Faculty of Engineering and Natural Sciences, 7840 Tampere University, 33720 Tampere, Finland; § Finnish Customs, P.O. Box 512, FI-00101 Helsinki, Finland; ∥ Department of Chemistry, University of Helsinki, 00014 Helsinki, Finland

## Abstract

This study explores pesticide detection with diverse ionization reagents by employing Multischeme chemical IONization inlet (MION) in conjunction with high-resolution Orbitrap mass spectrometry (MS). Various ionization schemes, specifically charging by Br^–^ and O_2_^–^ in negative polarity, and by H_3_O^+^ and C_3_H_6_OH^+^ in positive polarity, were investigated. The findings build on our previous work concerning pesticide detection using multischeme ionization and further demonstrate the effectiveness of the MION-MS methodology for detecting pesticides from complex standard mixtures and fruit extracts. The method successfully detected 136 compounds at a concentration of 10 ng/mL, and 447 at a concentration of 100 ng/mL, from standard solutions containing altogether 651 pesticides. The analysis of 10 fruit extracts revealed detections comparable to those obtained with validated methods. Subsequent molecular modeling provided insight into product identities observed when using protonated acetone as a reagent ion, which revealed that fragmentation into protonated pesticide and neutral acetone is energetically favored over decomposition to pesticide and protonated acetone (reactants). The current study amply underscores the versatility of the MION-MS methodology in seamlessly transitioning between different reagent ions in both polarities, enabling detection of a wider range of chemical compounds than with any single-ion-scheme instrument.

## Graphical abstract

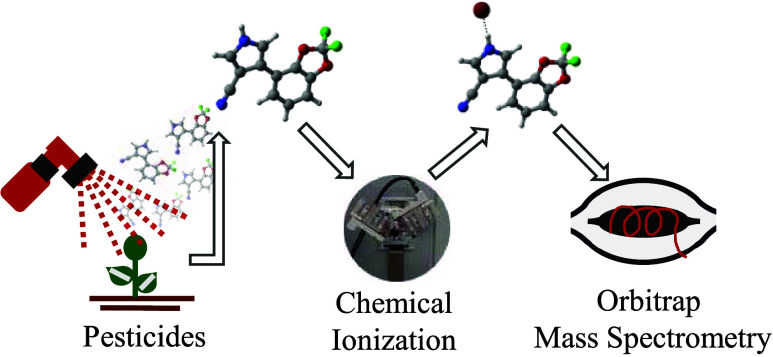

## Introduction

Pesticides play a pivotal role in modern agriculture. They safeguard crops against pests and diseases, and enhance productivity to meet global food demand.^1,2^ While many types of chemicals end up in the environment from a multitude of pollution sources, pesticides raise a considerable concern as they are applied directly in large quantities to the products of the food chain, particularly by the agricultural industry.^3^ Ideally, the applied pesticides should effectively eliminate the targeted pests without harming nontargeted species, including humans. However, this is often not the case, and only less than 1% of the utilized chemicals reach their targets.^4,5^

Pesticide exposure stems from various direct and indirect sources. The direct sources include occupational, agricultural, and household uses, whereas indirect pesticide exposure occurs through environmental media such as air, water, soil, and the food chain.^6,7^ The primary entry routes into the human body are dermal absorption, ingestion, and inhalation.^6^ Exposure to pesticides, whether through direct contact, handling, or residues in food, can lead to various health problems. The most common health consequences include oxidative stress, diabetes mellitus,^9^ respiratory disorders,^7^ neurological disorders,^8^ reproductive issues,^1^ and cancer^9–11^

Pesticides span a very wide range of chemical identities and, consequently, pose a considerable challenge for quantification. Accordingly, there is a growing trend to develop more diverse methods for measuring a wider collection of pesticides simultaneously.^12–14^ Additionally, different techniques are generally required for quantifying pesticides from different environmental media^14–16^ making new comprehensive evaluation methods crucial for mitigating risks associated with pesticide residues.^17^ Stringent monitoring of pesticide levels in foods and goods is imperative to safeguard public health and ensure regulatory compliance.

Pesticide residues in food samples are quantified with or without prior sample extraction. Various common extraction techniques employed include liquid–liquid extraction (LLE), supercritical fluid extraction (SFE), microwave-assisted extraction (MAE), solid-phase extraction (SPE), solid-phase microextraction (SPME), stir-bar sorptive extraction (SBSE), and QuEChERS (Quick, Easy, Cheap, Effective, Rugged, and Safe) extraction. Following the extraction step, the most common techniques used to quantify pesticides and their residues in foods include gas chromatography (GC), gas chromatography–mass spectrometry (GC-MS), high-performance liquid chromatography (HPLC), and liquid chromatography–mass spectrometry (LC-MS).^18–20^ In contrast, methods that do not require extraction have also been developed, such as desorption electrospray ionization mass spectrometry (DESI-MS),^21^ direct analysis in real-time mass spectrometry (DART-MS),^22^ paper spray mass spectrometry (PS-MS),^23^ and low-temperature plasma mass spectrometry (LTP-MS).^24^ Some methods such as atmospheric pressure chemical ionization (APCI)^25–27^ and atmospheric pressure photoionization mass spectrometry (APPI-MS)^28,29^ may or may not require an extraction step prior to analysis, depending on the nature of the sample.

Atmospheric pressure interface chemical ionization mass spectrometry (API-CIMS) is a powerful methodology for detecting organic and inorganic compounds in the gas phase.^30^ Due to its versatility, sensitivity, and robustness, it has become the tool of choice for the atmospheric science community in both field deployments and detailed laboratory investigations.^31–33^ Already in some of the early CIMS studies both positive and negative polarity chemical ionization was used to detect pesticides in environmental substrates^34^ and food samples,^35^ and the application of CIMS in pesticide detection has continued to the present time^36–38^ In this study, the multischeme chemical ionization inlet (MION)^39^ coupled to orbitrap mass spectrometry (MS) was used to investigate pesticide detection from standard mixtures and authentic fruit extracts using four unique ionization schemes including bromide (Br^–^), superoxide (O_2_^–^), protonated acetone (C_3_H_6_OH^+^), and hydronium (H_3_O^+^) ionization. To provide more insight into the target molecule and reagent ion interaction, the ionization by protonated acetone and bromide was further inspected using quantum chemical computations. The results of the work advance our understanding of gas- and condensed-phase pesticide detection and the general applicability of API-CIMS as a tool for quantifying the current state of the environment.

## Materials and Methods

### Instrumentation

The laboratory investigations employed a MION coupled with an upgraded LTQ (Linear Trap Quadrupole) Velos Pro orbitrap mass spectrometer from Thermo Fisher, operated at a resolution of 100,000. The ion injection time (IT) was set to 2 s with microscan set at one, and data were acquired over a mass range of 100–1000 *m*/*z*. The S-lens RF level was maintained at 60%, the capillary temperature was maintained at 100 °C, and both the sheath gas and auxiliary gas flow rates were kept at 1 arbitrary unit. Regarding ion optics, the multipole RF amplitude was approximately 795 Vp-p for both positive and negative ionization modes. The gate lens voltage was approximately −92 V in positive mode and 92 V in negative mode.

The experimental setup consisted of a thermal desorption (TD) unit (Karsa Ltd.) with a filter holder and an injection port positioned upstream of the MION system coupled to the MS (i.e., TD-MION-MS). The MION was operated with parallel Br^–^, O_2_^–^, C_3_H_6_OH^+^, and H_3_O^+^ ion schemes.^40^

All of the experiments utilized liquid samples which were introduced into the TD-MION-MS via syringe injections. Custom-made desorption filters (i.e., adsorbent-coated metal mesh filters, 37 mm in diameter, Karsa Ltd.) and a 10 μL Trajan Scientific SGE 10FX-5C syringe were used. The samples were desorbed for a gas-phase CIMS analysis by ramping the temperature from 30 to 250 °C.

The fruit extracts were analyzed at the Finnish Customs laboratories by using two complementary techniques. Gas chromatography–tandem mass spectrometry (GC-MS/MS) was performed with an Agilent 7890B GC system coupled to a 7010B triple quadrupole mass spectrometer (HES, MS/MS). The system was equipped with a multimode inlet (MMI) and two HP-5MS UI columns (15 m × 0.25 mm) configured with a BackFlush. Ionization was carried out using electron ionization (EI) at 70 eV, and data were acquired in multiple reaction monitoring (MRM) mode. In parallel, ultrahigh-performance liquid chromatography–tandem mass spectrometry (UHPLC-MS/MS) was conducted using a Waters UPLC Xevo TQ-XS system. Separation was achieved using an Acquity UPLC BEH C18 column (1.7 μm, 2.1 mm × 100 mm) with a VanGuard precolumn (2.1 mm × 5 mm). Electrospray ionization (ESI) was used in both positive and negative modes, with detection also occurring in the MRM mode.

### Reagents

Reagent ions were generated using direct X-ray irradiation (4.9 keV Hamamatsu L12536): dibromomethane (DBrMe) in negative polarity yielded bromide ions (Br^–^), acetone in positive polarity formed protonated acetone (C_3_H_6_OH^+^), and dried, purified ambient air (Purafil Charcoal scrubber, Ecotech) in both polarities provided hydronium (H_3_O^+^) and superoxide ions (O_2_^–^), respectively. The presence of trace amounts of water facilitated H_3_O^+^ production for proton transfer reactions, while oxygen from the air feed served as the O_2_^–^ source. Our analysis aimed to identify bromide adducts, deprotonated species formed due to the presence of superoxide ions, and protonated species generated by reactions with hydronium and protonated acetone reagent ions.

### Samples

Solution “A” and solution “B”, both provided by GALAB Laboratories GmbH in Hamburg, Germany, consisted of 369 and 300 pesticide standards, respectively. Acetonitrile was used as the solvent in the original solutions, as well as for dilution and washing. The complete list of pesticides, including compound names, CAS identifiers, and SMILES, is provided in Table S1 (Supporting Information). The full data set, available in the Zenodo database,^41^ details each compound’s peak area across specified concentrations (10, 20, 100, 1000, and 2500 ng/mL) and ionization methods. Reagent ions include bromide, protonated acetone, hydronium ions, and dioxide.

A total of 10 fruit and vegetable samples were analyzed, including grapefruit, lemon, two types of orange (orange A and orange B), bell pepper, two types of pineapple (pineapple A and pineapple B), spinach, strawberry, and tomato, along with their respective fruit extracts and information regarding the pesticides contained in each sample, were received from Finnish Customs. The extracts had been prepared by homogenizing with a Retsch GM300 grinder using dry ice from which 10 g were subjected to extraction using 10 mL of acetonitrile. To aid in the extraction process, a salting-out mixture, comprising 1 g of sodium chloride, 1 g of sodium citrate, and 0.5 g of sodium hydrogen citrate, was introduced. After vigorous shaking and centrifugation, the extract was purified using PSA (primary secondary amine) and MgSO_4_ (magnesium sulfate). Post-centrifugation, the purified extract was filtered into a glass vial. All sample preparation and analysis procedures were strictly adhered to the CEN EN 15662 standard.

To investigate the impact of other compounds present in the fruit matrix, a fig extract was spiked with solution “A”. Preparing the spiked sample involved combining 90 μL of fig extract (with no detected pesticides) with 10 μL of 1000 ng/mL solution “A”. The resulting solution (i.e., spiked fig extract) contained 100 ng/mL of pesticides.

### Workflow

The pesticide content of each sample was analyzed by placing the filter into a thermal desorber. Following this setup, sample solutions were injected onto the filter through a syringe injection port. The thermal desorption process involved a temperature ramp, beginning at 30 °C and rising to 250 °C in 48 s, with the maximum temperature maintained for the remainder of the approximately 4 min measurement duration.

Solutions “A” and “B” were analyzed using TD-MION-MS, with two distinct injection volumes, namely, 1 and 10 μL. With 1 μL volume, we investigated six distinct concentrations, which were 10, 20, 100, 200, 1000, and 2500 ng/mL. To minimize potential memory effects, only the three lowest concentrations were investigated with a volume of 10 μL (e.g., 10, 20, and 100 ng/mL).

For further studies, an 8 μL injection volume was selected. In previous measurements, it had been observed that injecting 10 μL volumes maintained a uniform thermal desorption. However, when working with extracts, 1 μL of acetonitrile was loaded into the syringe needle before drawing the sample. This hindered our ability to inject a 10 μL sample as the syringe capacity was limited to 10 μL. These considerations guided the sample volume selection.

The matrix effect was investigated by injecting 8 μL of spiked fig extract. The measurements were conducted by using the Br^–^ ion scheme. Four repetitions were performed.

### Data Analysis

Data analysis was conducted using the TraceFinder General Quan 4.1 software (Thermo Fisher), which employed the quantitation method using a compound database. The compound databases were manually customized to include the specific compounds being investigated in solution “A” and “B”. Within this method, parameters were set with a mass threshold of 5 ppm, a signal-to-noise ratio of 2, and a fit threshold of 80%.

The calibration curve was generated using data from separately injected calibration standards, solution A and solution B, at concentrations of 10, 20, 50, and 100 ng/mL. The software used these measurements to generate calibration curves, which were subsequently applied to estimate target concentrations.

### Quantum Chemical Computations

The stabilities of ionic adducts of dimefox, cycloate, trietazine, and crimidine with C_3_H_6_OH^+^, along with their respective tendencies to protonate, were examined using computational quantum chemical methods. A systematic conformational search was done using the MMFF molecular mechanics method in the Spartan ’20 program (Wavefunction Inc.). Single-point energies were computed at the B3LYP/6–31+G* level^42–44^ for all conformers using Spartan ’20, and those within 5 kcal/mol in electronic energies of the lowest-energy conformer were considered for geometry optimizations. Geometry optimizations were first carried out at the B3LYP/6–31+G* level of theory and subsequently at ωB97X-D/6–31+G* (with frequency calculations)^45^ for the conformers within 2 kcal·mol^–1^ in electronic energies of the lowest-energy conformer. These geometry optimization and frequency calculations were performed with the Gaussian 16 program.^46,47^

Protonation at all possible sites of the target pesticides (e.g., carbonyl, hydroxyl, amine, and halogen groups) was systematically checked, and those with the lowest relative enthalpies were used to study the energetics of the protonation channels we report. The number of hydrogen bond acceptor (HBA) and hydrogen bond donor (HBD) sites in detected pesticides was analyzed to explore potential correlations between these parameters and the selectivity between reagent ions. This analysis included pesticides detected as bromide adducts and as protonated, using acetone as a reagent, in solutions ″A″ and ″B”. Solutions were measured at a 20 ng/mL concentration with a 10 μL injection volume. The analysis was performed using ChemDraw (version 20.1.1, PerkinElmer, Inc.), which calculates HBD and HBA based on 2D molecular structures.

## Results and Discussion

### Standard Solutions Measurement

The experimental data reveal the unique characteristics of each ion scheme and polarity mode, producing specific mass spectra profiles. Figure 1 presents these profiles for hydronium (H_3_O^+^), protonated acetone (C_3_H_6_OH^+^), bromide (Br^–^), and superoxide (O_2_^–^) ions, derived from a 1000 ng/mL injection of solution “A” with spectra captured around 90 s into the measurement (100–500 *m*/*z*). Certain compounds detectable in one ion scheme remain undetected by others.

**Figure 1:**
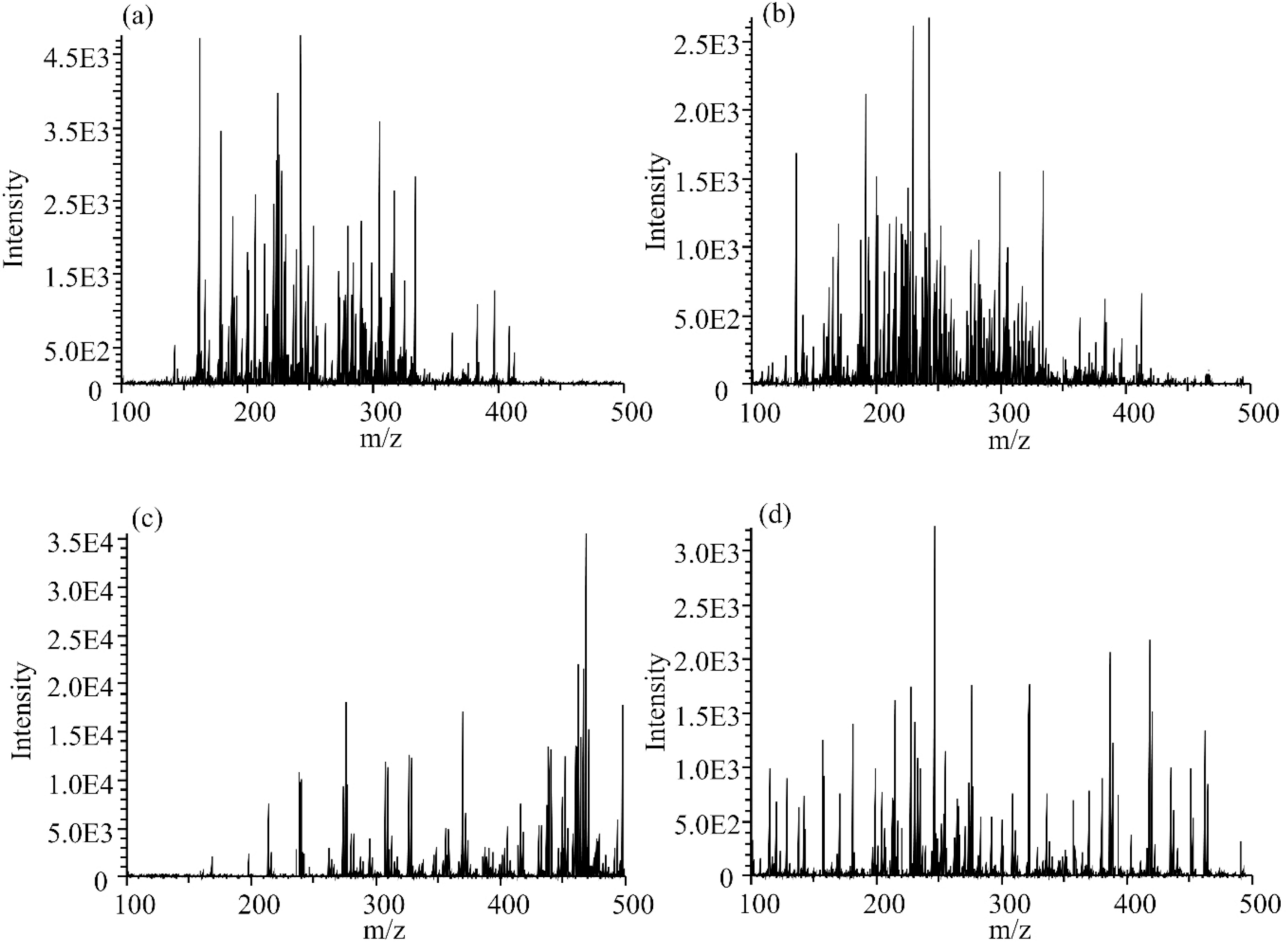
Characteristic spectra obtained from 369 pesticides with each ionization scheme: (a) hydronium (H_3_O^+^), (b) protonated acetone (C_3_H_6_OH^+^), (c) bromide (Br^–^), and (d) superoxide (O_2_^–^). Spectra were recorded from a 1000 ng/mL injection of solution A, captured at approximately 90 s into the measurement at 100–500 *m*/*z*.

Number of detected pesticides in solutions “A” and “B” at different concentrations (10, 20, 100, 200, 1000, and 2500 ng/mL) and volumes (1 and 10 μL) using Br^–^, H_3_O^+^, O_2_^–^, and C_3_H_6_OH^+^ ion schemes is summarized in Figure 2. A detailed tabulation of these results is available in Tables S1 and S2 of the Supporting Information.

**Figure 2:**
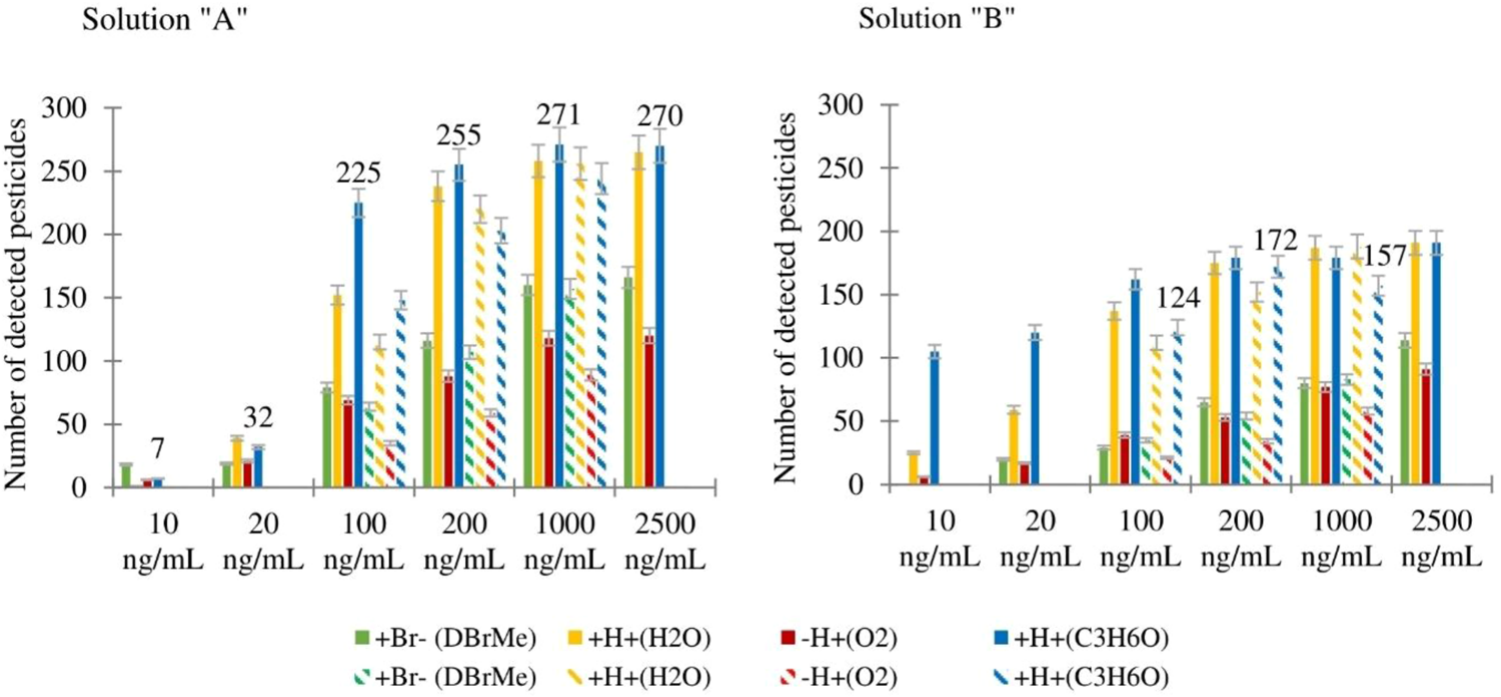
Result of different volumes of injection for solution “A” and solution “B” with all ionization schemes (the neutral reagent used is presented in brackets). Solid fill columns and pattern fill columns correspond to 1 μL injection volume and 10 μL injection volume, respectively. (The notation ″+Br–″ indicates the addition of bromide anion, leading to the formation of a bromide adduct.). Detection trends vary by reagent, with nonlinear scaling observed at higher loading amounts. The error bars indicate an estimated 5% uncertainty.

The results validate the expected pattern wherein an increase in concentration correlates with a higher number of detected pesticides, as the ionization efficiencies differ.^48^ This trend is particularly pronounced for concentrations at or below 100 ng/mL. Furthermore, the results illustrate a decrease in the number of detected pesticides with increasing sample concentration when using protonated acetone as a reagent for solution “B” with a 10 μL injection volume (pattern fill columns in the solution “B” graph). This decrease could also, at least partly, be attributed to human error, as the injections were performed manually for each ion scheme. On the other hand, a plateau pattern is observed for the same ion scheme, but in solution “A” with a 1 μL injection volume (see Figure 2). The number of detected pesticides does not increase linearly with an increasing amount of sample. Loading amounts beyond approximately 1 ng (i.e., comparing 100 ng/mL at 10 μL versus 1000 ng/mL at 1 μL injections) do not necessarily result in the detection of additional pesticides (see Figure S1 in the Supporting Information)

As previously stated, concentrations of 100, 200, and 1000 ng/mL were investigated in both 1 and 10 μL volumes (Figure 2)

Upon closer examination of the results, comparing the same ionization schemes with different volumes (solid filled columns and their corresponding pattern-filled columns in Figure 2), it appears that in most of the cases, the solvent does not significantly influence the results. This observation stems from experiments where the same amount of pesticides was injected but with varying volumes of solvent (e.g., 1 μL versus 10 μL injection volumes). The data suggest that the amount of the sample rather than the volume of the solvent used is the determining factor for the detection results. From the 651 pesticides, 447 pesticides were detected at a concentration of 100 ng/mL, 218 pesticides at 20 ng/mL, and 136 pesticides at 10 ng/mL.

Figure 3 illustrates the statistical results obtained from measurements at concentrations of 10, 20, and 100 ng/mL. It shows that the combination of Br^–^ and C_3_H_6_OH^+^ ionizations detects more pesticides than the combination of Br^–^ and the hydronium ion scheme. On the other hand, the results from the Br^–^ ionization scheme and the C_3_H_6_OH^+^ ion scheme combined achieve reasonably similar detection coverage compared to combining results from all applied ion schemes. This suggests that a sufficient level of information can be obtained, even with a subset of the reagent ion combinations. The latter subset of ionization schemes (e.g., Br^–^ ion attachment and protonation by C_3_H_6_OH^+^) was further utilized in this study.

**Figure 3:**
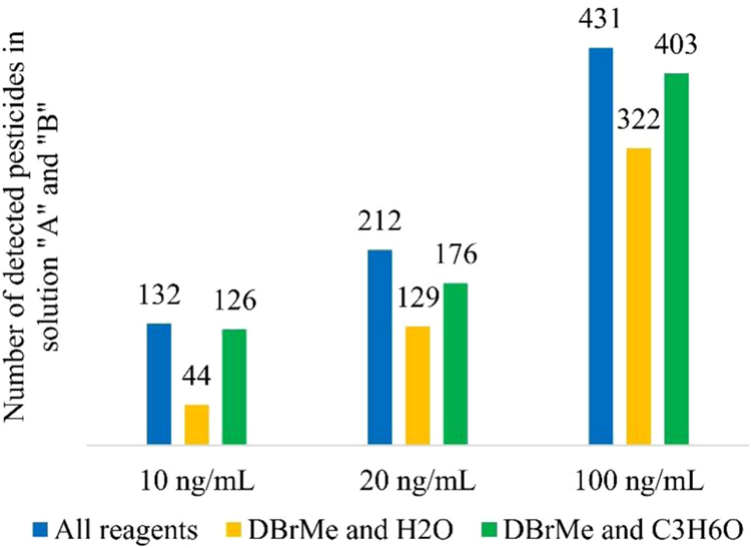
Statistical results for different pesticide concentrations using all reagents, combination of Br^–^ ion scheme and hydronium ion scheme, and combination of Br^–^ ion scheme and protonation with acetone as reagent.

### Fig Extract Matrix Measurement

A total of 96% of the compounds present in Solution A were detected in the spiked fig extract, demonstrating similar detection from a standard solution and a real-world matrix. Additionally, the average desorption peak area (i.e., the average intensity of detection) of the spiked fig extract was comparable to what was obtained with the direct injection of solution “A”. As an example, the detection of triflumuron at a concentration of 100 ng/mL in both the spiked extract and standard solution “A” is shown in Figure 4, with maximum relative intensities of 1521.87 and 1864.22, respectively, and an identical peak area of 29,400.

**Figure 4:**
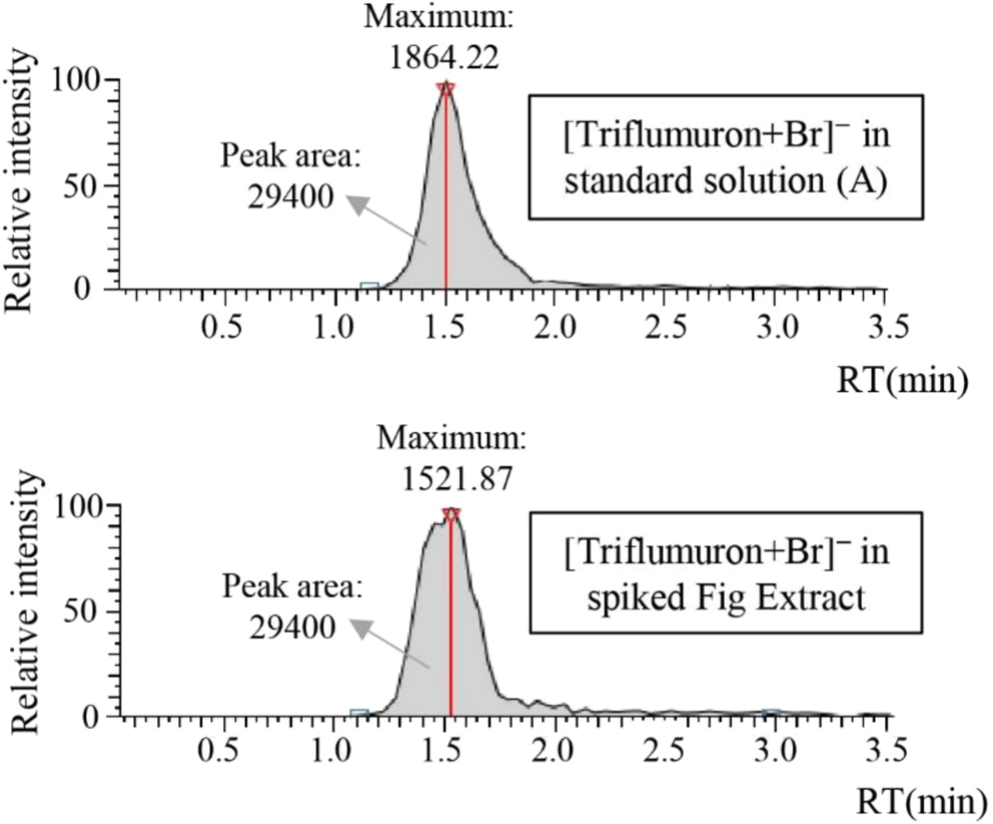
Triflumuron, at a concentration of 100 ng/mL, was detected in both the spiked extract and standard solution “A”.

### Extract Measurements

Measurements were conducted on 10 fruit and vegetable extracts using an 8 μL sample volume and 1 μL syringe washing solution (acetonitrile). The estimated concentrations of pesticides were then compared with the concentration data from Finnish Customs for each extract. The detailed outcomes are presented in Table 1. The full list of pesticides detected in each extract by Finnish Customs is presented in Table S4 in the Supporting Information.

**Table 1:** Pesticide Detection and Quantification in Fruit Extracts

			+Br– (DBrMe)		+H+ (C_3_H_6_O)	
sample	detected pesticides	^a^customs (μg/mL)	isotopic pattern (%)	^b^quantitative result (μg/mL)	isotopic pattern (%)	^b^quantitative result (μg/mL)
grapefruit	sulfoxaflor	11	100	20.5	100	84.3
	methoxyfenozide	14.1	0	20.4	0	11.2
	pyraclostrobin	14.1			0	25.4
	imazalil	400.9			0	345.1
lemon	pyriproxyfen	11.8			5	6.8
	pyraclostrobin	21.2			0	50.1
	carbendazim	58.2	0	31.1	100	36.3
	thiabendazole	636.7	0	19.1	100	475.4
	pyrimethanil	699.5			100	1073.1
	imazalil	786			100	1207.1
orange A	trifloxystrobin	20.4			16	19.5
	pyrimethanil	1179			58	1415.1
	thiabendazole	1179	0	28.7	61	624.6
	imazalil	1650.6			81	4440.7
orange B	propiconazol	9.4			0	7.4
	trifloxystrobin	11.8			15	16.4
	fenpyroximate	18.8			0	32.2
	thiabendazole	864.6	0	34.4	84	556
	imazalil	1414.8			97	2708
	pyrimethanil	1807.8			85	1715.3
bell pepper	acetamiprid	7.9				
	azoxystrobin	8.6			1	18.4
	imidacloprid	16.5			0	19.8
pineapple A	diazinon	8.6			98	17.5
	fludioxonil	377.3	100	242.7	100	86.8
pineapple B	fludioxonil	235.8	100	172.48		
spinach	pyraclostrobin	172.9			33	2261.7
	boscalid	1886.4	92	360.7	48	35.5
strawberry	ethirimol	12.6	0	30.4	66	46.9
	penconazole	17.3	0	7.65	0	5.1
	clofentezine	18.1				
	pyraclostrobin	26.7			19	63.7
	spinosad	62.1				
	trifloxystrobin	125.7			15	661.1
	fluopyram	157.2	80	70.6	0	696.5
	boscalid	172.9	0	25.1	62	12.6
	fludioxonil	314.4	100	202	100	143.6
	cyprodinil	377.3			63	626.1
tomato	fluopyram	16.5	30	15.4	36	48.3

a Quantified by the Finnish custom’s laboratory.

b Results from this study.

After reviewing Table 1, differences in detected pesticide numbers between the Br^–^ ionization and protonation by C_3_H_6_OH^+^ are evident. Specifically, protonation resulted in the detection of more pesticides. This finding corroborates the information illustrated in Figure 2, which indicates that protonation with C_3_H_6_OH^+^ as the ionization method yields the highest number of detections overall compared to the other reagents.

Fludioxonil was consistently detected in extracts (e.g., pineapple sample A, pineapple sample B, and strawberry) when using Br^–^ ionization. Interestingly, protonated acetone was able to retrieve fluodioxonil from strawberry and pineapple A but not from the pineapple B extract (Table 1). Fludioxonil was also well detected in our previous study.^48^ All other compounds detected with bromide ionization were also detected with protonated acetone. These results, along with the observed cross-sensitivities, stem from the structural complexity of pesticides, which often possess both donor and acceptor sites. As a result, they can interact with multiple reagent ions and be detected across different polarities

The results obtained using the TD-MION-MS setup were compared to the quantitative findings by Finnish Customs, shown in Table 1. In certain cases, such as fluopyram in tomato (Br^–^ ionization), imidacloprid in bell pepper, trifloxystrobin in orange A, propiconazol in orange B (all with C_3_H_6_OH^+^ ionization), and methoxyfenozide in grapefruit (both ionizations), the concentration estimates were accurate. However, discrepancies were observed with trifloxystrobin in strawberry (C_3_H_6_OH^+^ ionization), thiabendazole in orange A (both ionizations), and pyraclostrobin (C_3_H_6_OH^+^ ionization) and boscalid (both ionizations) in spinach. It is important to note that the Customs’ concentration results were estimated using external standard measurements performed within the same fruit extraction matrix as the actual samples. In contrast, for this investigation, the concentration estimation was based on external standard data obtained from standard solutions prepared in pure acetonitrile. Additionally, the Customs’ results were obtained from freshly prepared extracts, while the results of this study were obtained after the extracts have been stored in a freezer for six months. The type and variety of fruit also often influences the quantitative outcome, even postsolvent extraction,^49^ as demonstrated by the detection of fludioxonil in strawberry and one pineapple variety (Pineapple A extract), but not in the other variety (Pineapple B extract).

### Quantum Chemically Derived Molecular Adduct Characteristics

The stabilities of the M­(C_3_H_6_OH^+^) adducts were computationally studied, where M denotes the pesticides cycloate, dimefox, trietazine, and crimidine. For each pesticide, the energies required for the fragmentation of the adducts into protonated pesticides (MH^+^) and neutral acetone, as well as the energies needed for the decomposition back into the reactants (M + C_3_H_6_OH^+^), were calculated (see Figure 5). The molecular structures of these compounds with their various protonation channels are presented in the Supporting Information as Figure S2a–d.

**Figure 5:**
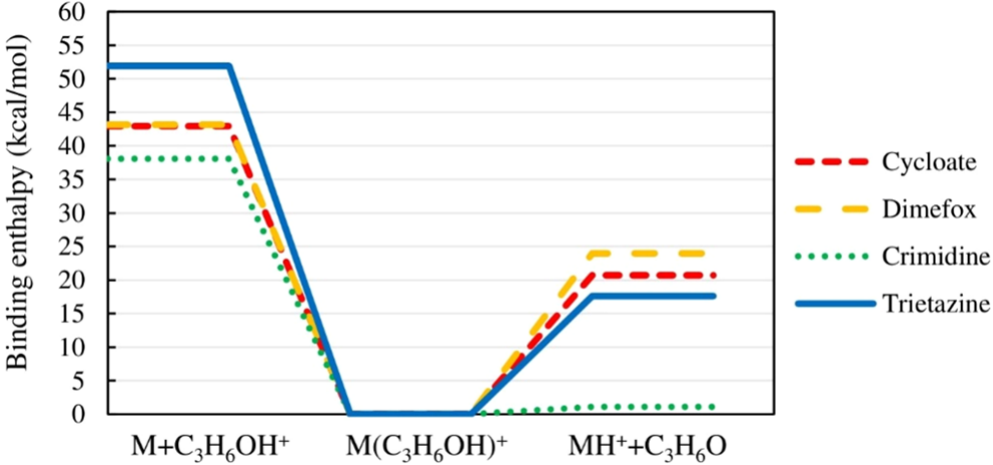
Computed enthalpy of formation energies suggest fragmentation of cycloate, dimefox, trietazine, and crimidine adducts with protonated acetone toward protonated pesticide (MH^*+*^).

The fragmentation of adducts toward protonated pesticides is more favorable in all four cases with corresponding adduct binding enthalpies of dimefox: 23.98 kcal/mol, cycloate: 20.72 kcal/mol, trietazine: 17.61 kcal/mol, and crimidine: 1.07 kcal/mol. The determined proton affinities of all four pesticides are higher than those of acetone, which aligns well with the observed preference for protonated pesticide formation during the experiments. However, the computed binding enthalpies do not correlate with the measured signal strengths, indicating that the detection sensitivity of the current desorption approach results from more factors than just the enthalpy of the CIMS reagent ion binding and the subsequent protonation reaction. Thus, also the target pesticide vapor pressures were compared, as they could infer potential sample transfer limitations in these gas-sampling measurements. However, consideration of the sample vapor pressures did not appear to improve the situation, again indicating that more factors are involved. Table 2 presents the results of the computations and selects previously reported values including calculated binding energies, proton affinities, vapor pressures, and experimentally measured peak areas

**Table 2:** Calculated Energies, Proton Affinities, Peak Areas, and Vapor Pressures of Crimidine, Cycloate, Dimefox, and Trietazine^a^

name	^b^binding enthalpy (kcal/mol)	^c^binding enthalpy (kcal/mol)	binding enthalpy difference	^d^proton affinity (kcal/mol)	^e^vapor pressure (Pa at 25 °C)	peak area
crimidine	38.09	1.07	37.02	230.78	3.07	62,300
trietazine	51.94	17.61	34.33	228.09	0.01	62,300
cycloate	42.94	20.72	22.22	215.98	0.23	92,200
dimefox	43.17	23.98	19.19	212.95	48	89,200
acetone				194.83 (^f^194.41)	^f^30,600	

a The calculated proton affinity and vapor pressure of acetone are included as a reference for comparison.

b [M­(C_3_H_6_OH^+^) → M + C_3_H_6_OH^+^].

c [M­(C_3_H_6_OH^+^) → MH^+^ + C_3_H_6_O].

d Values from the current work.

e Vapor pressure values for each pesticide were retrieved from the PubChem database, using the entries reported at 25 °C. For cycloate, two available values were found, and their average was used.^50–54^

f Proton affinity and vapor pressure values for acetone from the NIST Chemistry WebBook. Proton affinity was averaged from the values reported at 25 °C, while vapor pressure was calculated using the Antoine equation parameters listed under phase change data.^55^

Iyer et al.^30^ showed previously that a threshold binding enthalpy of about 26 kcal/mol exists for an iodide time-of-flight (TOF) CIMS, where adducts more strongly bound undergo negligible fragmentation. Conversely, less strongly bound adducts undergo fragmentation more readily at scales proportional to the deficit from the above threshold. The calculated binding enthalpies of cycloate, dimefox, trietazine, and crimidine adducts are below the 26 kcal/mol threshold and are observed as protonated (the mass spectral window for the four compounds is provided in Figure S3 in the Supporting Information). In the case of dimefox, the formation of an adduct could potentially be expected; however, it was not observed in the measurements conducted.

In our previous study, eight pesticides forming Br^–^ adducts showed a near-linear correlation between computed adduct formation enthalpies and detection sensitivity.^48^ Building on these findings, in this study, we conducted a computational analysis focused on pesticides capable of forming adducts with Br^–^. The calculated adduct formation enthalpies were plotted against experimental detection sensitivity, represented by the integrated peak area (PA) under the thermal desorption profile normalized by vapor pressure. However, a similar linear correlation was not observed in the current results, potentially indicating problems with the normalization procedure or more subtle factors than simple binding enthalpy controlling the detection sensitivity between the bromide ion and the complex pesticide targets. The corresponding graph and computational results can be found in Figure S4 and Table S6 in the Supporting Information.

The number of hydrogen bond acceptor (HBA) and donor (HBD) sites was analyzed for pesticides detected via Br^–^ ionization and C_3_H_6_OH^+^ ionization. In a previous study, the relationship between HBA and HBD for pesticides detected via Br^–^ ionization was explored.^48^ To enable a direct comparison between these ionization schemes, this study presents the peak area of the desorption profile plotted against the imbalance of acceptor and donor sites (HBA minus HBD) for both Br^–^ and C_3_H_6_OH^+^ ionization (Figure 6).

**Figure 6:**
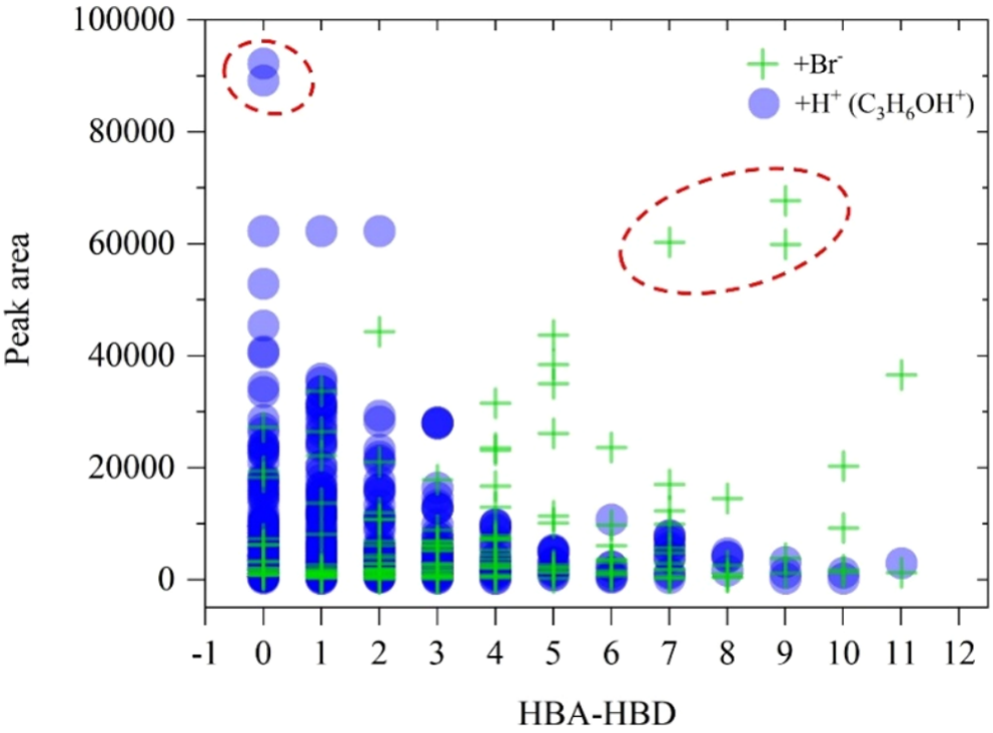
Relationship between peak area and the imbalance of acceptor (HBA) and donor (HBD) sites for both protonated (by C3H6OH^+^ ionization scheme: blue circles) and bromide adducts (green crosses). Regions with the largest peak areas are circled in red, highlighting protonated cycloate and dimefox via C_3_H_6_OH^+^ ionization and bromide adducts of fipronil-desulfinyl, fipronil-sulfide, and fluxapyroxad.

Protonated species tend to have a smaller imbalance between acceptor and donor sites while having larger peak areas. On the other hand, bromide adducts, which show larger peak areas, have a greater difference when subtracting the number of HBD sites from the number of HBA sites. This could indicate that the formation efficiency of bromide adducts may be influenced by the availability of hydrogen bond acceptor (HBA) and hydrogen bond donor (HBD) sites. In contrast, protonation by C_3_H_6_OH^+^ may rely more on direct protonation interactions, resulting in their comparatively large peak areas despite a lower HBA-HBD disparity. Protonated cycloate and dimefox via C_3_H_6_OH^+^ ionization, along with bromide adducts of fipronil-desulfinyl, fipronil-sulfide, and fluxapyroxad, exhibit the highest peak areas among the analyzed pesticides (highlighted with red dotted circles in Figure 6).

## Conclusions

This study highlights the effectiveness of the integrated Multischeme chemical IONization inlet (MION) combined with high-resolution Orbitrap mass spectrometry (MS) for detecting pesticides, investigating 651 pesticides in standard solutions and 10 fruit extracts. Experimental findings indicate that different reagent ions and polarities yield unique mass spectra, facilitating comprehensive pesticide detection. The utilization of all ionization schemes employed (negative mode: bromide (Br^–^) clustering and deprotonation by superoxide ions (O_2_^–^) from ambient air oxygen; positive mode: protonation by protonated acetone (C_3_H_6_OH^+^) and by hydronium ions (H_3_O^+^, from water in ambient air)) resulted in the detection of 447 pesticides at a concentration of 100 ng/mL, 218 pesticides at 20 ng/mL, and 136 pesticides at 10 ng/mL, out of the total 651 pesticides. It is noteworthy that combining results from the Br^–^ ionization scheme and the C_3_H_6_OH^+^ ion scheme achieves nearly equivalent detection coverage as combining results from all applied ion schemes.

Comparing the results obtained using the MION-MS setup and those from Finnish Customs using validated detection methods highlights the potential of the developed methodology. Differences in detected pesticide numbers are evident, with protonation by C_3_H_6_OH^+^ detecting more pesticides than Br^–^ ionization. Despite variations from factors such as sample storage, fruit type, and the use of acetonitrile solvent instead of the exact fruit extract matrix for external standard injections, concentration estimates were accurate in certain cases. Examples include fluopyram in tomato extract with Br^–^ chemical ionization and imidacloprid in bell pepper extract with C_3_H_6_OH^+^ ionization.

The stabilities of ionic adducts of cycloate, dimefox, trietazine, and crimidine with C_3_H_6_OH^+^ and their propensities to protonate were studied using computational quantum chemical methods. Their fragmentation channel toward protonated pesticide MH^+^ and neutral acetone is more energetically favorable than decomposition back to the reactants M + C_3_H_6_OH^+^.

The use of various reagent ions and polarities improves detection coverage significantly, as no single reagent can detect all pesticides. Despite some variability due to the experimental conditions, the results highlight the reliability and potential of MION-MS for pesticide detection across a range of concentrations and matrices.

## Supplementary Material



## Data Availability

Quantum chemical output files are available online through the public research data archive Zenodo (https://doi.org/10.5281/zenodo.14414608).

## Notes

Full list of all the pesticides under study including the isomers; detection results for solution “A” and “B” across different ionization schemes and concentrations; diagrams showing the different protonation channels for trietazine, crimidine, cycloate and dimefox; and illustration of calculated cluster formation enthalpies versus detection sensitivity (integrated peak area, PA, normalized by vapor pressure) for pesticides forming Br^–^ adducts and their values of cluster formation enthalpies (kcal/mol), vapor pressure at 20 °C (mPa), peak area under the desorption profile, and peak area normalized by vapor pressure (PDF)
