# Supplementary material for: Ambient-Pressure Multischeme Chemical Ionization for Pesticide Detection: A MION-Orbitrap Mass Spectrometry Study
Source: ACS Omega. 2025 May 23;10(21):21324–33. doi: 10.1021/acsomega.4c11287 (PMC12138600; doi:10.1021/acsomega.4c11287)
Supplement: Supplementary file 1 [file ao4c11287_si_001.pdf]

Ambient Pressure Multi-Scheme Chemical Ionization for Pesticide Detection: A MION-Orbitrap Mass Spectrometry Study

Fariba Partovi[1,2], Joona Mikkilä[1,*], Siddharth Iyer[2], Jyri Mikkilä[1], Jussi Kontro[1], Suvi Ojanperä[3], Aleksei Shcherbinin[1], Matti Rissanen[2,4,*]

[1]Karsa Ltd., A. I. Virtasen aukio 1, 00560 Helsinki, Finland

[2]Aerosol Physics Laboratory, Physics Unit, Faculty of Engineering and Natural Sciences, Tampere University, 33720 Tampere, Finland

[3]Finnish Customs, P.O. Box 512, FI-00101 Helsinki, Finland

[4]Department of Chemistry, University of Helsinki, 00014 Helsinki, Finland

Table S1. The full list of all pesticides including all isomers.

Name	CAS	SMILES
Captafol	2425-06-1	ClC(Cl)C(Cl)(Cl)SN1C(=O)C2CC=CCC2C1=O
Chinomethionate	2439-01-2	Cc1ccc2nc3SC(=O)Sc3nc2c1
Chloropropylate	5836-10-2	CC(C)OC(=O)C(C1=CC=C(C=C1)Cl)(C2=CC=C(C=C2)Cl)O
Dodin	2439-10-3	CCCCCCCCCCCCN=C(N)N.CC(O)=O
Fenthion oxon	6552-12-1	CO[P](=O)(OC)Oc1ccc(SC)c(C)c1
Lenacil	2164-08-1	O=C1NC2=C(CCC2)C(=O)N1C3CCCCC3
Methiocarb-Sulfoxide	2635-10-1	CNC(=O)Oc1cc(C)c(c(C)c1)[S](C)=O
Phenthoate	2597-03-7	CCOC(=O)C(C1=CC=CC=C1)SP(=S)(OC)OC
Phorat-Oxon-Sulfon	2588-06-9	CCOP(=O)(OCC)SCS(=O)(=O)CC
Phorate sulfone	2588-04-7	CCOP(=S)(OCC)SCS(=O)(=O)CC
Phorate sulfoxide	2588-03-6	CCOP(=S)(OCC)SCS(=O)CC
1,4-Dimethylnaphthalene	571-58-4	Cc1ccc(C)c2ccccc12
1-Naphthylacetamide	86-86-2	NC(=O)Cc1cccc2ccccc12
1-Naphthylacetamide	86-86-2	NC(=O)Cc1cccc2ccccc12
1-Naphthaleneacetic acid	86-87-3	OC(=O)Cc1cccc2ccccc12
2,4,5-T	93-76-5	OC(=O)COc1cc(Cl)c(Cl)cc1Cl
2,4,5-T-Methylester	1928-37-6	COC(=O)COC1=CC(=C(C=C1Cl)Cl)Cl
2,4-D	94-75-7	OC(=O)COc1ccc(Cl)cc1Cl
2,4-DB	94-82-6	OC(=O)CCCOc1ccc(Cl)cc1Cl
2,4-D-Methylester	1928-38-7	COC(=O)COC1=C(C=C(C=C1)Cl)Cl
2,4-DP	120-36-5	CC(Oc1ccc(Cl)cc1Cl)C(O)=O
3,4,5-Trimethacarb	2686-99-9	CNC(=O)Oc1cc(C)c(C)c(C)c1
3-Decen-2-one	18402-84-1	CCCCCCC=CC(C)=O
4-Bromophenylurea	1967-25-5	NC(=O)Nc1ccc(Br)cc1
4-Chloro-3-methylphenol	59-50-7	Cc1cc(O)ccc1Cl
4-CPA	122-88-3	OC(=O)COc1ccc(Cl)cc1

Abamectin B1a	65195-55-3	CCC(C)C1C(C=CC2(O1)CC3CC(O2)CC=C(C(C(C=CC=C4COC5C4(C(C=C(C5O)C)C(=O)O3)O)C)OC6CC(C(C(O6)C)OC7CC(C(C(O7)C)O)OC)OC)C)C
Acephate	30560-19-1	COP(=O)(NC(C)=O)SC
Acetamiprid	160430-64-8	CN(Cc1ccc(Cl)nc1)C(C)=NC#N
Acetochlor	34256-82-1	CCOCN(C(=O)CCl)c1c(C)cccc1CC
Acibenzolar acid	35272-27-6	OC(=O)c1cccc2nnsc12
Acibenzolar acid	35272-27-6	C1=CC(=C2C(=C1)N=NS2)C(=O)S
Acibenzolar-S-methyl	135158-54-2	CSC(=O)c1cccc2nnsc12
Aclonifen	74070-46-5	Nc1c(Cl)c(Oc2ccccc2)ccc1[N+]([O-])=O
Acrinathrin	101007-06-1	CC1(C)[C@@H](\C=C/C(=O)OC(C(F)(F)F)C(F)(F)F)[C@H]1C(=O)O[C@H](C#N)c2cccc(Oc3ccccc3)c2
Alachlor	15972-60-8	CCc1cccc(CC)c1N(COC)C(=O)CCl
Aldicarb	116-06-3	CNC(ON=CC(C)(C)SC)=O
Aldicarb-sulfoxide	1646-87-3	CNC(ON=CC(C)(C)S(C)=O)=O
Aldoxycarb	1646-88-4	CNC(=O)O\N=C\C(C)(C)[S](C)(=O)=O
Aldrin	309-00-2	ClC1=C(Cl)C2(Cl)C3C4CC(C=C4)C3C1(Cl)C2(Cl)Cl
Allethrin	584-79-2	C\C(C)=C/C1C(C(=O)OC2CC(=O)C(CC=C)=C2C)C1(C)C
Ametoctradin	865318-97-4	CCCCCCCCc1c(CC)nc2cnnn2c1N
Ametryn	834-12-8	CCNc1nc(NC(C)C)nc(SC)n1
Amidithion	919-76-6	COCCNC(=O)CS[P](=S)(OC)OC
Amidosulfuron	120923-37-7	COc1cc(OC)nc(NC(=O)N[S](=O)(=O)N(C)[S](C)(=O)=O)n1
Aminocarb	2032-59-9	CNC(=O)Oc1ccc(N(C)C)c(C)c1
Amisulbrom	348635-87-0	CN(C)S(=O)(=O)n1cnc(n1)S(=O)(=O)n2c(C)c(Br)c3ccc(F)cc23
Amitraz	33089-61-1	CN(\C=N\c1ccc(C)cc1C)\C=N\c2ccc(C)cc2C
Ancymidol	12771-68-5	COc1ccc(cc1)C(O)(C2CC2)c3cncnc3

Anilazin	101-05-3	Clc1nc(Cl)nc(Nc2ccccc2Cl)n1
Anthraquinone	84-65-1	O=C1c2ccccc2C(=O)c3ccccc13
Atrazine	1912-24-9	CCNc1nc(Cl)nc(NC(C)C)n1
Atrazine-desethyl	6190-65-4	CC(C)Nc1nc(N)nc(Cl)n1
Azaconazol	60207-31-0	C1COC(O1)(CN2C=NC=N2)C3=C(C=C(C=C3)Cl)Cl
Azadirachtin	11141-17-6	[H]C1([H])C2OC3([H])OC=CC3(O)C1C4(C)OC24[C@@]5(C)[C@@H](O)[C@@H]6OC[C@]7([C@H](C[C@H](OC(=O)C(C)=CC)[C@]8(COC(O)([C@H]58)C(=O)OC)[C@@]67[H])OC(C)=O)C(=O)OC
Azamethiphos	35575-96-3	CO[P](=O)(OC)SCN1C(=O)Oc2cc(Cl)cnc12
Azinphos ethyl	2642-71-9	CCO[P](=S)(OCC)SCN1N=Nc2ccccc2C1=O
Azinphos-Methyl	86-50-0	COP(=S)(OC)SCN1N=Nc2ccccc2C1=O
Aziprotryne	4658-28-0	CSc1nc(NC(C)C)nc(N=[N+]=[N-])n1
Azoxystrobin	131860-33-8	CO\C=C(\C(=O)OC)c1ccccc1Oc2cc(Oc3ccccc3C#N)ncn2
Stearyldimethylbenzyl ammonium chloride	122-19-0	CCCCCCCCCCCCCCCCCC[N+](C)(C)CC1=CC=CC=C1.[Cl-]
Barban	101-27-9	ClCC#CCOC(=O)Nc1cccc(Cl)c1
Benalaxyl	71626-11-4	COC(=O)C(C)N(C(=O)Cc1ccccc1)c2c(C)cccc2C
Bendiocarb	22781-23-3	CNC(=O)Oc1cccc2OC(C)(C)Oc12
Benfluralin	1861-40-1	CCCCN(CC)c1c(cc(cc1[N+]([O-])=O)C(F)(F)F)[N+]([O-])=O
Benfuracarb	82560-54-1	CCOC(=O)CCN(SN(C)C(=O)Oc1cccc2CC(C)(C)Oc12)C(C)C
Benodanil	15310-01-7	Ic1ccccc1C(=O)Nc2ccccc2
Benomyl	17804-35-2	CCCCNC(=O)n1c(NC(=O)OC)nc2ccccc12
Bensulfuron-Methyl	83055-99-6	COC(=O)c1ccccc1C[S](=O)(=O)NC(=O)Nc2nc(OC)cc(OC)n2
Bentazon	25057-89-0	CC(C)N1C(=O)c2ccccc2NS1(=O)=O

6-Hydroxy Bentazon	60374-42-7	CC(C)N1C(=O)C2=C(C=CC(=C2)O)NS1(=O)=O
Bentazone-8-hydroxy	60374-43-8	CC(C)N1C(O)c2cccc(O)c2NS1(=O)=O
Benthiavalicarb isopropyl	177406-68-7	CC(C)OC(=O)N[C@@H](C(C)C)C(=O)N[C@H](C)c1nc2ccc(F)cc2s1
Benzoylprop-Ethyl	22212-55-1	CCOC(=O)C(C)N(C(=O)c1ccccc1)c2ccc(Cl)c(Cl)c2
Benzyladenin	1214-39-7	Nc1nc(Cc2ccccc2)nc3nc[nH]c13
Benzyldimethyldecylammonium chlorid	965-32-2	[Cl-].CCCCCCCCCC[N+](C)(C)Cc1ccccc1
Benzyldimethyldodecylammonium chlorid	139-07-1	[Cl-].CCCCCCCCCCCC[N+](C)(C)Cc1ccccc1
Benzyldimethylhexadecyl ammonium chlorid	122-18-9	[Cl-].CCCCCCCCCCCCCCCC[N+](C)(C)Cc1ccccc1
Benzyldimethyloctylammonium	46917-11-7	CCCCCCCC[N+](C)(C)CC1=CC=CC=C1
Benzyldimethyloctylammonium chloride	959-55-7	[Cl-].CCCCCCCC[N+](C)(C)Cc1ccccc1
Benzyldimethyltetradecylammonium chloride	139-08-2	CCCCCCCCCCCCCC[N+](C)(C)CC1=CC=CC=C1.[Cl-]
Bifenazate	149877-41-8	COc1ccc(cc1NNC(=O)OC(C)C)-c2ccccc2
Bifenox	42576-02-3	COC(=O)c1cc(Oc2ccc(Cl)cc2Cl)ccc1[N+]([O-])=O
Bifenthrin	99267-18-2	CC1(C)C(C(OCC2=C(C)C(C3=CC=CC=C3)=CC=C2)=O)C1/C=C(Cl)/C(F)(F)F
Binapacryl	485-31-4	CCC(C)c1cc(cc(c1OC(=O)C=C(C)C)[N+]([O-])=O)[N+]([O-])=O
Biphenyl	92-52-4	c1ccc(cc1)-c2ccccc2
Bispyribac	125401-75-4	COC1=CC(=NC(=N1)OC2=C(C(=CC=C2)OC3=NC(=CC(=N3)OC)OC)C(=O)O)OC
Bitertanol	55179-31-2	CC(C)(C)C(O)C(Oc1ccc(cc1)-c2ccccc2)n3cncn3
Bixafen	581809-46-3	Cn1cc(C(=O)Nc2ccc(F)cc2-c3ccc(Cl)c(Cl)c3)c(n1)C(F)F
Boscalid	188425-85-6	Clc1ccc(cc1)-c2ccccc2NC(=O)c3cccnc3Cl

Bromacil	314-40-9	CCC(C)N1C(=O)NC(=C(Br)C1=O)C
Bromfenvinphos	33399-00-7	CCO[P](=O)(OCC)O\C(=C\Br)c1ccc(Cl)cc1Cl
Bromocyclen	1715-40-8	C1C(C2(C(=C(C1(C2(Cl)Cl)Cl)Cl)Cl)Cl)CBr
Bromophos-ethyl	4824-78-6	CCOP(=S)(OCC)Oc1cc(Cl)c(Br)cc1Cl
Bromophos	2104-96-3	CO[P](=S)(OC)Oc1cc(Cl)c(Br)cc1Cl
Bromoxynil	1689-84-5	Oc1c(Br)cc(cc1Br)C#N
Bromoxynil heptanoate	56634-95-8	CCCCCCC(=O)OC1=C(C=C(C=C1Br)C#N)Br
Bromoxynil-methyl ether	3336-39-8	COC1=C(C=C(C=C1Br)C#N)Br
Bromoxynil Octanoate	1689-99-2	CCCCCCCC(=O)OC1=C(C=C(C=C1Br)C#N)Br
Bromopropylate	18181-80-1	CC(C)OC(=O)C(O)(c1ccc(Br)cc1)c2ccc(Br)cc2
Bromuconazol	116255-48-2	Clc1ccc(c(Cl)c1)C2(CC(Br)CO2)Cn3cncn3
Bupirimate	41483-43-6	CCCCc1c(C)nc(NCC)nc1OS(=O)(=O)N(C)C
Buprofezin	69327-76-0	CC(C)N1C(=O)N(CS\C1=N\C(C)(C)C)c2ccccc2
Butachlor	23184-66-9	CCCCOCN(C(=O)CCl)c1c(CC)cccc1CC
Butafenacil	134605-64-4	CN1C(=O)N(C(=O)C=C1C(F)(F)F)c2ccc(Cl)c(c2)C(=O)OC(C)(C)C(=O)OCC=C
Butoxycarboxim	34681-23-7	CNC(=O)O\N=C(/C)C(C)[S](C)(=O)=O
Butralin	33629-47-9	CCC(C)Nc1c(cc(cc1[N+]([O-])=O)C(C)(C)C)[N+]([O-])=O
Buturon	3766-60-7	CC(C#C)N(C)C(=O)Nc1ccc(Cl)cc1
Cadusafos	95465-99-9	CCO[P](=O)(SC(C)CC)SC(C)CC
Captan	133-06-2	ClC(Cl)(Cl)SN1C(=O)C2CC=CCC2C1=O
Carbaryl	63-25-2	CNC(=O)Oc1cccc2ccccc12
Carbendazim	10605-21-7	COC(=O)Nc1nc2ccccc2[nH]1
Carbetamid	16118-45-9	CCNC(=O)C(C)OC(=O)NC1=CC=CC=C1
Carbofuran	1563-66-2	CNC(=O)Oc1cccc2CC(C)(C)Oc12
Carbofuran-3-hydroxy	16655-82-6	CNC(=O)Oc1cccc2C(O)C(C)(C)Oc12

Carbophenothion	786-19-6	CCO[P](=S)(OCC)SCSc1ccc(Cl)cc1
Methyl trithion	953-17-3	CO[P](=S)(OC)SCSc1ccc(Cl)cc1
Carbosulfan	55285-14-8	CCCCN(CCCC)SN(C)C(=O)Oc1cccc2CC(C)(C)Oc12
Carboxine	5234-68-4	CC1=C(SCCO1)C(=O)Nc2ccccc2
Carboxin Sulfoxide	17757-70-9	CC1=C(S(=O)CCO1)C(=O)NC2=CC=CC=C2
Carfentrazone-ethyl	128639-02-1	CCOC(=O)C(Cl)Cc1cc(N2N=C(C)N(C(F)F)C2=O)c(F)cc1Cl
Chlorbensid	103-17-3	Clc1ccc(CSc2ccc(Cl)cc2)cc1
Chlorbenzilat	510-15-6	CCOC(=O)C(O)(c1ccc(Cl)cc1)c2ccc(Cl)cc2
Chlorbromuron	13360-45-7	CON(C)C(=O)Nc1ccc(Br)c(Cl)c1
Chlorbufam	1967-16-4	CC(OC(=O)Nc1cccc(Cl)c1)C#C
cis-Chlordane	5103-71-9	C1[C@H]2[C@@H]([C@H]([C@H]1Cl)Cl)[C@]3(C(=C([C@@]2(C3(Cl)Cl)Cl)Cl)Cl)Cl
trans-Chlordane	5103-74-2	C1[C@H]2[C@@H]([C@H]([C@@H]1Cl)Cl)[C@]3(C(=C([C@@]2(C3(Cl)Cl)Cl)Cl)Cl)Cl
Chlordecone	143-50-0	ClC1(Cl)C2(Cl)C3(Cl)C4(Cl)C(=O)C5(Cl)C3(Cl)C1(Cl)C5(Cl)C24Cl
Chlorfenapyr	122453-73-0	CCOCn1c(-c2ccc(Cl)cc2)c(C#N)c(Br)c1C(F)(F)F
Chlorfenprop-Methyl	14437-17-3	COC(=O)C(Cl)Cc1ccc(Cl)cc1
Chlorfenson	80-33-1	Clc1ccc(O[S](=O)(=O)c2ccc(Cl)cc2)cc1
Chlorfenvinphos	470-90-6	CCOP(=O)(OCC)O\C(=C\Cl)c1ccc(Cl)cc1Cl
Chlorfluazuron	71422-67-8	Fc1cccc(F)c1C(=O)NC(=O)Nc2cc(Cl)c(Oc3ncc(cc3Cl)C(F)(F)F)c(Cl)c2
Chloridazon	1698-60-8	NC1=C(Cl)C(=O)N(N=C1)c2ccccc2
CHLORIDAZON-DESPHENYL	6339-19-1	C1=NNC(=O)C(=C1N)Cl
Chlormephos	24934-91-6	CCO[P](=S)(OCC)SCCl
Chloroneb	2675-77-6	COc1cc(Cl)c(OC)cc1Cl
Chloroxuron	1982-47-4	CN(C)C(=O)Nc1ccc(Oc2ccc(Cl)cc2)cc1
Chlorpropham	101-21-3	CC(C)OC(=O)Nc1cccc(Cl)c1

Chlorpyrifos	2921-88-2	CCOP(=S)(OCC)Oc1nc(Cl)c(Cl)cc1Cl
Chlorpyrifos-Methyl	5598-13-0	COP(=S)(OC)Oc1nc(Cl)c(Cl)cc1Cl
Chlorsulfuron	64902-72-3	COc1nc(C)nc(NC(=O)N[S](=O)(=O)c2cccc c2Cl)n1
Chlorthal-Dimethyl	1861-32-1	COC(=O)c1c(Cl)c(Cl)c(c(Cl)c1Cl)C(=O)OC
Chlorthalonil	1897-45-6	Clc1c(Cl)c(C#N)c(Cl)c(C#N)c1Cl
Chlorthiamid	1918-13-4	NC(=S)c1c(Cl)cccc1Cl
Chlorthion	500-28-7	CO[P](=S)(OC)Oc1ccc(c(Cl)c1)[N+]([O-])=O
Chlorthiophos	60238-56-4	CCO[P](=S)(OCC)Oc1cc(Cl)c(SC)cc1Cl
Chlortoluron	15545-48-9	CN(C)C(=O)Nc1ccc(C)c(Cl)c1
Chlozolinate	84332-86-5	CCOC(=O)C1(C)OC(=O)N(C1=O)c2cc(Cl) cc(Cl)c2
Cinerin I	25402-06-6	CC=CCC1=C(C(CC1=O)OC(=O)C2C(C2(C)C)C=C(C)C)C
Cinidon-Ethyl	142891-20-1	CCOC(=O)\C(Cl)=C\c1cc(ccc1Cl)N2C(=O) C3=C(CCCC3)C2=O
Cinosulfuron	94593-91-6	COCCOc1ccccc1[S](=O)(=O)NC(=O)Nc2nc (OC)nc(OC)n2
cis-1,2,3,6-Tetrahydrophthalimide	1469-48-3	[H][C@]12CC=CC[C@@]1([H])C(=O)NC2 =O
Clethodim	99129-21-2	CCC(=NOCC=CCl)C1=C(CC(CC1=O)CC(C)SCC)O
Clethodim sulfone	111031-17-5	CCC(=NOCC=CCl)C1=C(CC(CC1=O)CC(C)S(=O)(=O)CC)O
Clethodim sulfoxide	111031-14-2	CCC(=NOCC=CCl)C1=C(CC(CC1=O)CC(C)S(=O)CC)O
Climbazol	38083-17-9	CC(C)(C)C(=O)C(Oc1ccc(Cl)cc1)n2ccnc2
Clodinafop Propargyl	105512-06-9	CC(C(=O)OCC#C)OC1=CC=C(C=C1)OC2 =C(C=C(C=N2)Cl)F
Clofentezine	74115-24-5	C1=CC=C(C(=C1)C2=NN=C(N=N2)C3=C C=CC=C3Cl)Cl
Clomazon	81777-89-1	CC1(CON(C1=O)CC2=CC=CC=C2Cl)C
Clopyralid	1702-17-6	OC(=O)c1nc(Cl)ccc1Cl
Cloquintocet-Mexyl	99607-70-2	CCCCCC(C)OC(=O)COc1ccc(Cl)c2cccnc12

Clothianidin	210880-92-5	CN=C(NCc1sc(Cl)nc1)N[N+]([O-])=O
Coumaphos	56-72-4	CCO[P](=S)(OCC)Oc1ccc2C(=C(Cl)C(=O)Oc2c1)C
Crimidin	535-89-7	CN(C)c1cc(C)nc(Cl)n1
Crufomat	299-86-5	CN[P](=O)(OC)Oc1ccc(cc1Cl)C(C)(C)C
Cyanazin	21725-46-2	CCNc1nc(Cl)nc(NC(C)(C)C#N)n1
Cyanofenphos	13067-93-1	CCO[P](=S)(Oc1ccc(cc1)C#N)c2ccccc2
Cyanophos	2636-26-2	CO[P](=S)(OC)Oc1ccc(cc1)C#N
Cyantraniliprole	736994-63-1	CC1=CC(=CC(=C1NC(=O)C2=CC(=NN2C3=C(C=CC=N3)Cl)Br)C(=O)NC)C#N
Cyazofamid	120116-88-3	CN(C)[S](=O)(=O)n1c(nc(Cl)c1c2ccc(C)cc2)C#N
Cyclanilide	113136-77-9	C1CC1(C(=O)NC2=C(C=C(C=C2)Cl)Cl)C(=O)O
Cycloate	1134-23-2	CCSC(=O)N(CC)C1CCCCC1
Cycloxydim	101205-02-1	CCCC(=NOCC)C1=C(CC(CC1=O)C2CCCSC2)O
Cyflufenamid	180409-60-3	C1CC1CON=C(C2=C(C=CC(=C2F)F)C(F)(F)F)NC(=O)CC3=CC=CC=C3
Cyflumetofen	400882-07-7	CC(C)(C)C1=CC=C(C=C1)C(C#N)(C(=O)C2=CC=CC=C2C(F)(F)F)C(=O)OCCOC
Cyfluthrin	68359-37-5	CC1(C)C(C=C(Cl)Cl)C1C(=O)OC(C#N)c2ccc(F)c(Oc3ccccc3)c2
Cyhalofop-butyl	122008-85-9	CCCCOC(=O)[C@@H](C)Oc1ccc(Oc2ccc(cc2F)C#N)cc1
lambda-Cyhalothrin	91465-08-6	CC1(C)[C@@H](\C=C(/Cl)C(F)(F)F)[C@H]1C(=O)OC(C#N)c2cccc(Oc3ccccc3)c2
Cyhexatin	13121-70-5	C1CCC(CC1)[Sn](C2CCCCC2)C3CCCCC3.O
Cymiazol	61676-87-7	CN1C=CSC1=Nc2ccc(C)cc2C
Cymoxanil	57966-95-7	CCNC(=O)NC(=O)\C(=N\OC)C#N
Cypermethrin	52315-07-8	CC1(C)C(C=C(Cl)Cl)C1C(=O)OC(C#N)c2cccc(Oc3ccccc3)c2

alpha-Cypermethrin	67375-30-8	CC1(C)C(\C=C(\Cl)Cl)C1C(=O)OC(C#N)c2cccc(Oc3ccccc3)c2
Cyphenothrin	39515-40-7	CC(C)=CC1C(C(=O)OC(C#N)c2cccc(Oc3ccccc3)c2)C1(C)C
Cyproconazol	94361-06-5	CC(C1CC1)C(CN2C=NC=N2)(C3=CC=C(C=C3)Cl)O
Cyprodinil	121552-61-2	Cc1cc(nc(Nc2ccccc2)n1)C3CC3
Cyprofuram	69581-33-5	Clc1cccc(c1)N(C2CCOC2=O)C(=O)C3CC3
Cyromazin	66215-27-8	Nc1nc(N)nc(NC2CC2)n1
Daminozide	1596-84-5	CN(C)NC(=O)CCC(O)=O
Dazomet	533-74-4	CN1CSC(=S)N(C)C1
Didecyldimethylammonium chloride	7173-51-5	[Cl-].CCCCCCCCCC[N+](C)(C)CCCCCCCCCC
Mitotane	53-19-0	ClC(Cl)C(c1ccc(Cl)cc1)c2ccccc2Cl
p,p'-DDD	72-54-8	ClC(Cl)C(c1ccc(Cl)cc1)c2ccc(Cl)cc2
o,p'-DDE	3424-82-6	Clc1ccc(cc1)C(=C(Cl)Cl)c2ccccc2Cl
p,p'-DDE	72-55-9	Clc1ccc(cc1)C(=C(Cl)Cl)c2ccc(Cl)cc2
o,p'-DDT	789-02-6	Clc1ccc(cc1)C(c2ccccc2Cl)C(Cl)(Cl)Cl
p,p'-DDT	50-29-3	Clc1ccc(cc1)C(c2ccc(Cl)cc2)C(Cl)(Cl)Cl
DEF	78-48-8	CCCCSP(=O)(SCCCC)SCCCC
Deltamethrin	52918-63-5	CC1(C)[C@@H](C=C(Br)Br)[C@H]1C(=O)O[C@H](C#N)c2cccc(Oc3ccccc3)c2
Metaisoseptox	919-86-8	CCSCCS[P](=O)(OC)OC
Demeton-S-methylsulphon	17040-19-6	CC[S](=O)(=O)CCS[P](=O)(OC)OC
Denatonium Benzoate	3734-33-6	CC[N+](CC)(CC1=CC=CC=C1)CC(=O)NC2=C(C=CC=C2C)C.C1=CC=C(C=C1)C(=O)[O-]
Deisopropylatrazine	1007-28-9	CCNC1=NC(=NC(=N1)N)Cl
Desmedipham	13684-56-5	CCOC(=O)Nc1cccc(OC(=O)Nc2ccccc2)c1
Desmetryn	1014-69-3	CNc1nc(NC(C)C)nc(SC)n1
Diafenthiuron	80060-09-9	CC(C)c1cc(Oc2ccccc2)cc(C(C)C)c1NC(=S)NC(C)(C)C
Dialifos	10311-84-9	CCO[P](=S)(OCC)SC(CCl)N1C(=O)c2ccccc2C1=O

Diallate	2303-16-4	CC(C)N(C(C)C)C(=O)SC\C(Cl)=C\Cl
Diazinon	333-41-5	CCO[P](=S)(OCC)Oc1cc(C)nc(n1)C(C)C
Dicamba	1918-00-9	COc1c(Cl)ccc(Cl)c1C(O)=O
Dichlobenil	1194-65-6	Clc1cccc(Cl)c1C#N
Dichlofenthion	97-17-6	CCO[P](=S)(OCC)Oc1ccc(Cl)cc1Cl
Dichlofluanid	1085-98-9	CN(C)[S](=O)(=O)N(SC(F)(Cl)Cl)c1ccccc1
Dichlorvos	62-73-7	CO[P](=O)(OC)OC=C(Cl)Cl
Diclobutrazol	75736-33-3	CC(C)(C)C(O)C(Cc1ccc(Cl)cc1Cl)n2cncn2
Diclofop	40843-25-2	CC(Oc1ccc(Oc2ccc(Cl)cc2Cl)cc1)C(O)=O
Diclofop methyl	51338-27-3	COC(=O)C(C)Oc1ccc(Oc2ccc(Cl)cc2Cl)cc1
Dicloran	99-30-9	Nc1c(Cl)cc(cc1Cl)[N+]([O-])=O
Dicofol	115-32-2	OC(c1ccc(Cl)cc1)(c2ccc(Cl)cc2)C(Cl)(Cl)Cl
Dicrotophos	141-66-2	CO[P](=O)(OC)OC(/C)=C/C(=O)N(C)C
Didodecyldimethylammonium Bromide	3282-73-3	[Br-].CCCCCCCCCCCC[N+](C)(C)CCCCCCCCCCCC
Dieldrin	60-57-1	C1[C@@H]2[C@H]3[C@@H]([C@H]1[C@H]4[C@@H]2O4)[C@]5(C(=C([C@@]3(C5(Cl)Cl)Cl)Cl)Cl)Cl
Diethofencarb	87130-20-9	CCOc1ccc(NC(=O)OC(C)C)cc1OCC
DEET	134-62-3	CCN(CC)C(=O)c1cccc(C)c1
Difenoconazol	119446-68-3	CC1COC(Cn2cncn2)(O1)c3ccc(Oc4ccc(Cl)cc4)cc3Cl
Difenoxuron	14214-32-5	COc1ccc(Oc2ccc(NC(=O)N(C)C)cc2)cc1
Diflubenzuron	35367-38-5	Fc1cccc(F)c1C(=O)NC(=O)Nc2ccc(Cl)cc2
Diflufenican	83164-33-4	Fc1ccc(NC(=O)c2cccnc2Oc3cccc(c3)C(F)(F)F)c(F)c1
Dimefox	115-26-4	CN(C)[P](F)(=O)N(C)C
Dimefuron	34205-21-5	CN(C)C(=O)Nc1ccc(N2N=C(OC2=O)C(C)(C)C)c(Cl)c1
Dimethachlor	50563-36-5	COCCN(C(=O)CCl)c1c(C)cccc1C
Dimethenamid	87674-68-8	COCC(C)N(C(=O)CCl)c1c(C)scc1C

Dimethipin	55290-64-7	CC1=C(C)[S](=O)(=O)CC[S]1(=O)=O
Dimethoate	60-51-5	CNC(=O)CS[P](=S)(OC)OC
Dimethomorph	110488-70-5	COc1ccc(cc1OC)C(=C\C(=O)N2CCOCC2)/ c3ccc(Cl)cc3
Dimethyldioctylammonium bromide	3026-69-5	CCCCCCCC[N+](C)(C)CCCCCCCC.[Br-]
Dimethylphenylformamide, 2.4-	60397-77-5	CC1=CC(=C(C=C1)NC=O)C
Dimetilan	644-64-4	CN(C)C(=O)Oc1cc(C)n(n1)C(=O)N(C)C
Dimoxystrobin	149961-52-4	CNC(=O)C(=N/OC)\c1ccccc1COc2cc(C)ccc 2C
Diniconazole	83657-24-3	CC(C)(C)C(C(=CC1=C(C=C(C=C1)Cl)Cl)N 2C=NC=N2)O
Dinitramin	29091-05-2	CCN(CC)C1=C(C=C(C(=C1[N+](=O)[O-])N)C(F)(F)F)[N+](=O)[O-]
Dinobuton	973-21-7	CCC(C)c1cc(cc(c1OC(=O)OC(C)C)[N+]([O -])=O)[N+]([O-])=O
Dinoseb	88-85-7	CCC(C)c1cc(cc(c1O)[N+]([O-])=O)[N+]([O-])=O
Dinoseb-Acetat	2813-95-8	CCC(C)C1=C(C(=CC(=C1)[N+](=O)[O-])[N+](=O)[O-])OC(=O)C
Dinotefuran	165252-70-0	CN=C(NCC1CCOC1)N[N+](=O)O
Dioxacarb	6988-21-2	CNC(=O)Oc1ccccc1C2OCCO2
Diphenamid	957-51-7	CN(C)C(=O)C(c1ccccc1)c2ccccc2
Diphenylamine	122-39-4	N(c1ccccc1)c2ccccc2
Dipropetryn	4147-51-7	CCSc1nc(NC(C)C)nc(NC(C)C)n1
Disulfoton	298-04-4	CCO[P](=S)(OCC)SCCSCC
Disulfoton-Sulfon	2497 06 5	CCOP(=S)(OCC)SCCS(=O)(=O)CC
Disulfoton-Sulfoxid	2497 07 6	CCOP(=S)(OCC)SCCS(=O)CC
Ditalimfos	5131-24-8	CCO[P](=S)(OCC)N1C(=O)c2ccccc2C1=O
Dithianon	3347-22-6	O=C1C2=C(SC(=C(S2)C#N)C#N)C(=O)c3c cccc13
Diuron	330-54-1	CN(C)C(=O)Nc1ccc(Cl)c(Cl)c1
DMSA	304-55-2	OC(=O)C(S)C(S)C(O)=O
DMST	66840-71-9	CN(C)[S](=O)(=O)Nc1ccc(C)cc1

DNOC	534-52-1	Cc1cc(cc(c1O)[N+]([O-])=O)[N+]([O-])=O
Dodecylguanidine	112-65-2	CCCCCCCCCCCCNC(N)=N
Dodemorph	1593-77-7	CC1CN(CC(C)O1)C2CCCCCCCCCCC2
Edifenphos	17109-49-8	CCO[P](=O)(Sc1ccccc1)Sc2ccccc2
Emamectin B1a	155569-91-8	CCC(C)C1C(C=CC2(O1)CC3CC(O2)CC=C(C(C(C=CC=C4COC5C4(C(C=C(C5O)C)C(=O)O3)O)C)OC6CC(C(C(O6)C)OC7CC(C(C(O7)C)NC)OC)OC)C)C
Empenthrin	54406-48-3	CC\C=C(/C)C(OC(=O)C1C(C=C(C)C)C1(C)C)C#C
alpha-Endosulfan	959-98-8	ClC1=C(Cl)C2(Cl)C3CO[S](=O)OCC3C1(Cl)C2(Cl)Cl
beta-Endosulfan	33213-65-9	ClC1=C(Cl)C2(Cl)[C@@H]3CO[S](=O)OC[C@@H]3C1(Cl)C2(Cl)Cl
Endosulfansulfate	1031-07-8	C1C2C(COS(=O)(=O)O1)C3(C(=C(C2(C3(Cl)Cl)Cl)Cl)Cl)Cl
Endrin	72-20-8	ClC1=C(Cl)C2(Cl)C3C4CC(C5OC45)C3C1(Cl)C2(Cl)Cl
Endrin ketone	53494-70-5	ClC1C2(Cl)C3C4CC5C3C(Cl)(C2(Cl)Cl)C1(Cl)C5C4=O
EPN	2104-64-5	CCO[P](=S)(Oc1ccc(cc1)[N+]([O-])=O)c2ccccc2
Epoxiconazol	133855-98-8	Fc1ccc(cc1)[C@@]2(Cn3cncn3)O[C@@H]2c4ccccc4Cl
EPTC	759-94-4	CCCN(CCC)C(=O)SCC
Esfenvalerate	66230-04-4	CC(C)[C@@H](C(=O)O[C@@H](C#N)c1cccc(Oc2ccccc2)c1)c3ccc(Cl)cc3
Etaconazol	71245-23-3	CCC1COC(Cn2cncn2)(O1)c3ccc(Cl)cc3Cl
Ethiofencarb	29973-13-5	CCSCc1ccccc1OC(=O)NC
Ethiofencarb-sulfon	53380-23-7	CCS(=O)(=O)Cc1ccccc1OC(=O)NC
Ethion	563-12-2	CCO[P](=S)(OCC)SCS[P](=S)(OCC)OCC
Ethirimol	245-949-3	CCCCC1=C(C)NC(=NC1=O)NCC
Ethofumesate	26225-79-6	CCOC1Oc2ccc(O[S](C)(=O)=O)cc2C1(C)C
Ethofumesate-2-keto	26244-33-7	CC1(C)C(=O)Oc2ccc(O[S](C)(=O)=O)cc12

Ethofumesate metabolite NC 20645	572912-13-1	CC(C)(C1=C(C=CC(=C1)OS(=O)(=O)C)O)C(=O)O
Ethoprophos	13194-48-4	CCCS[P](=O)(OCC)SCCC
Ethoxyquin	91-53-2	CCOc1ccc2NC(C)(C)C=C(C)c2c1
Ethylenethiourea	96-45-7	S=C1NCCN1
Etofenprox	80844-07-1	CCOc1ccc(cc1)C(C)(C)COCc2cccc(Oc3ccccc3)c2
Etoxazole	153233-91-1	CCOc1cc(ccc1C2COC(=N2)c3c(F)cccc3F)C(C)(C)C
Etridiazole	2593-15-9	CCOc1snc(n1)C(Cl)(Cl)Cl
Etrimfos	38260-54-7	CCOc1cc(O[P](=S)(OC)OC)nc(CC)n1
Famoxadone	131807-57-3	CC1(OC(=O)N(Nc2ccccc2)C1=O)c3ccc(Oc4ccccc4)cc3
Famphur	52-85-7	CO[P](=S)(OC)Oc1ccc(cc1)[S](=O)(=O)N(C)C
Fenamidone	161326-34-7	CC1(C(=O)N(C(=N1)SC)NC2=CC=CC=C2)C3=CC=CC=C3
Fenamiphos	22224-92-6	CCO[P](=O)(NC(C)C)Oc1ccc(SC)c(C)c1
Fenamiphos-Sulfone	31972-44-8	CCO[P](=O)(NC(C)C)Oc1ccc(c(C)c1)[S](C)(=O)=O
Fenamiphos-Sulfoxide	31972-43-7	CCO[P](=O)(NC(C)C)Oc1ccc(c(C)c1)[S](C)=O
Fenarimol	60168-88-9	OC(c1ccc(Cl)cc1)(c2cncnc2)c3ccccc3Cl
Fenazaquin	120928-09-8	CC(C)(C)c1ccc(CCOc2ncnc3ccccc23)cc1
Fenbuconazol	114369-43-6	Clc1ccc(CCC(Cn2cncn2)(C#N)c3ccccc3)cc1
Fenbutatin-oxide	13356-08-6	CC(C)(C[Sn](CC(C)(C)C1=CC=CC=C1)(CC(C)(C)C2=CC=CC=C2)O[Sn](CC(C)(C)C3=CC=CC=C3)(CC(C)(C)C4=CC=CC=C4)CC(C)(C)C5=CC=CC=C5)C6=CC=CC=C6
ethyl 1-(2,4-dichlorophenyl)-5-(trichloromethyl)-1,2,4-triazole-3-carboxylate	103112-35-2	CCOC(=O)c1nn(c2ccc(Cl)cc2Cl)c(n1)C(Cl)(Cl)Cl
Fenchlorphos	299-84-3	CO[P](=S)(OC)Oc1cc(Cl)c(Cl)cc1Cl
Fenchlorphos-oxon	3983-45-7	CO[P](=O)(OC)Oc1cc(Cl)c(Cl)cc1Cl
Fenhexamid	126833-17-8	CC1(CCCCC1)C(=O)Nc2ccc(O)c(Cl)c2Cl
Fenitrothion	122-14-5	CO[P](=S)(OC)Oc1ccc(c(C)c1)[N+]([O-])=O

Fenobucarb	3766-81-2	CCC(C)c1ccccc1OC(=O)NC
Fenoprop	93-72-1	CC(Oc1cc(Cl)c(Cl)cc1Cl)C(O)=O
Fenothiocarb	62850-32-2	CN(C)C(=O)SCCCCOc1ccccc1
Acclaim	66441-23-4	CCOC(=O)C(C)Oc1ccc(Oc2oc3cc(Cl)ccc3n2)cc1
Fenoxycarb	72490-01-8	CCOC(=O)NCCOc1ccc(Oc2ccccc2)cc1
Fenpiclonil	74738-17-3	Clc1cccc(c1Cl)c2c[nH]cc2C#N
Fenpicoxamid	517875-34-2	CC1C(C(C(=O)OCC(C(=O)O1)NC(=O)C2=NC=CC(=C2OCOC(=O)C(C)C)OC)CC3=CC=CC=C3)OC(=O)C(C)C
Fenpropathrin	39515-41-8	CC1(C)C(C(=O)OC(C#N)c2cccc(Oc3ccccc3)c2)C1(C)C
Fenpropidin	67306-00-7	CC(CN1CCCCC1)Cc2ccc(cc2)C(C)(C)C
Fenpropimorph	67564-91-4	CC(CN1CC(C)OC(C)C1)Cc2ccc(cc2)C(C)(C)C
Fenpyrazamine	473798-59-3	CC1=CC=CC=C1C2=C(N(N(C2=O)C(C)C)C(=O)SCC=C)N
Fenpyrazamine	473798-59-3	CC1=CC=CC=C1C2=C(N(N(C2=O)C(C)C)C(=O)SCC=C)N
Fenpyroximate	134098-61-6	Cn1nc(C)c(\C=N\OCc2ccc(cc2)C(=O)OC(C)(C)C)c1Oc3ccccc3
Fenson	80-38-6	Clc1ccc(O[S](=O)(=O)c2ccccc2)cc1
Fensulfothion	115-90-2	CCO[P](=S)(OCC)Oc1ccc(cc1)[S](C)=O
Fensulfothion Sulfone	14255-72-2	CCOP(=S)(OCC)OC1=CC=C(C=C1)S(=O)(=O)C
Fenthion	55-38-9	CO[P](=S)(OC)Oc1ccc(SC)c(C)c1
Fenthoxon Sulfoxide	6552-13-2	CC1=C(C=CC(=C1)OP(=O)(OC)OC)S(=O)C
Fenthion-sulfone	3761-42-0	CO[P](=S)(OC)Oc1ccc(c(C)c1)[S](C)(=O)=O
Fenthion-sulfoxide	3761-41-9	CC1=C(C=CC(=C1)OP(=S)(OC)OC)S(=O)C
Triphenyltin hydride	892-20-6	c1ccc(cc1)[Sn](c2ccccc2)c3ccccc3
Fenuron	101-42-8	CN(C)C(=O)Nc1ccccc1
Fipronil	120068-37-3	Nc1n(nc(C#N)c1[S](=O)C(F)(F)F)c2c(Cl)cc(cc2Cl)C(F)(F)F

Fipronil Desulfinyl	205650-65-3	C1=C(C=C(C(=C1Cl)N2C(=C(C(=N2)C#N)C(F)(F)F)N)Cl)C(F)(F)F
Fipronil-Sulfide	120067-83-6	Nc1n(nc(C#N)c1SC(F)(F)F)c2c(Cl)cc(cc2Cl)C(F)(F)F
Fipronil-Sulfone	120068-36-2	Nc1n(nc(C#N)c1[S](=O)(=O)C(F)(F)F)c2c(Cl)cc(cc2Cl)C(F)(F)F
Flamprop-M-Isopropyl	57973-67-8	CC(C)OC(=O)[C@H](C)N(C(=O)c1ccccc1)c2ccc(F)c(Cl)c2
methyl (2R)-2-(N-benzoyl-3-chloro-4-fluoroanilino)propanoate	63729-98-6	COC(=O)[C@@H](C)N(C(=O)c1ccccc1)c2ccc(F)c(Cl)c2
Flazasulfuron	104040-78-0	COc1cc(OC)nc(NC(=O)N[S](=O)(=O)c2ncccc2C(F)(F)F)n1
Flonicamid	158062-67-0	FC(F)(F)c1ccncc1C(=O)NCC#N
Fluazifop	69335-91-7	CC(Oc1ccc(Oc2ccc(cn2)C(F)(F)F)cc1)C(O)=O
Fluazifop-Butyl	69806-50-4	CCCCOC(=O)C(C)Oc1ccc(Oc2ccc(cn2)C(F)(F)F)cc1
Fluazifop-P-Butyl	79241-46-6	CCCCOC(=O)[C@@H](C)Oc1ccc(Oc2ccc(cn2)C(F)(F)F)cc1
Fluazinam	79622-59-6	[O-][N+](=O)c1cc(c(Cl)c(c1Nc2ncc(cc2Cl)C(F)(F)F)[N+]([O-])=O)C(F)(F)F
Fluazuron	86811-58-7	Fc1cccc(F)c1C(=O)NC(=O)Nc2ccc(Cl)c(Oc3ncc(cc3Cl)C(F)(F)F)c2
Flubendiamide	272451-65-7	Cc1cc(ccc1NC(=O)c2cccc(I)c2C(=O)NC(C)(C)C[S](C)(=O)=O)C(F)(C(F)(F)F)C(F)(F)F
Flubenzimin	37893-02-0	FC(F)(F)N=C1SC(=Nc2ccccc2)N(c3ccccc3)C1=NC(F)(F)F
Fluchloralin	33245-39-5	CCCN(CCCl)c1c(cc(cc1[N+]([O-])=O)C(F)(F)F)[N+]([O-])=O
Flucythrinat	70124-77-5	CC(C)C(C1=CC=C(C=C1)OC(F)F)C(=O)OC(C#N)C2=CC(=CC=C2)OC3=CC=CC=C3
Fludioxonil	131341-86-1	FC1(F)Oc2cccc(c2O1)c3c[nH]cc3C#N
Fluensulfone	318290-98-1	C1=C(SC(=N1)S(=O)(=O)CCC(=C(F)F)F)Cl
Flufenacet	142459-58-3	CC(C)N(C(=O)COc1sc(nn1)C(F)(F)F)c2ccc(F)cc2

Flufenacet oxalate	201668-31-7	CC(C)N(c1ccc(F)cc1)C(=O)C(O)=O
flufenacet ESA	201668-32-8	CC(C)N(C1=CC=C(C=C1)F)C(=O)CS(=O)(=O)O
2-[2-(4-fluoro-N-propan-2-ylanilino)-2-oxoethyl]sulfinylacetic acid	201668-33-9	CC(C)N(C1=CC=C(C=C1)F)C(=O)CS(=O)CC(=O)O
Flufenoxuron	101463-69-8	Fc1cc(Oc2ccc(cc2Cl)C(F)(F)F)ccc1NC(=O)NC(=O)c3c(F)cccc3F
Flumethrin	69770-45-2	CC1(C)C(\C=C(Cl)\c2ccc(Cl)cc2)C1C(=O)OC(C#N)c3ccc(F)c(Oc4ccccc4)c3
Flumetralin	62924-70-3	CCN(Cc1c(F)cccc1Cl)c2c(cc(cc2[N+]([O-])=O)C(F)(F)F)[N+]([O-])=O
Flumioxazin	103361-09-7	Fc1cc2OCC(=O)N(CC#C)c2cc1N3C(=O)C4=C(CCCC4)C3=O
Fluometuron	2164-17-2	CN(C)C(=O)Nc1cccc(c1)C(F)(F)F
Fluopicolide	239110-15-7	C1=CC(=C(C(=C1)Cl)C(=O)NCC2=C(C=C(C=N2)C(F)(F)F)Cl)Cl
Fluopyram	658066-35-4	C1=CC=C(C(=C1)C(=O)NCCC2=C(C=C(C=N2)C(F)(F)F)Cl)C(F)(F)F
Fluorodifen	15457-05-3	[O-][N+](=O)c1ccc(Oc2ccc(cc2[N+]([O-])=O)C(F)(F)F)cc1
Fluotrimazol	31251-03-3	FC(F)(F)c1cccc(c1)C(n2cncn2)(c3ccccc3)c4ccccc4
Fluoxastrobin	361377-29-9	CO\N=C(C1=NOCCO1)/c2ccccc2Oc3ncnc(Oc4ccccc4Cl)c3F
Flupyradifurone	951659-40-8	C1C(=CC(=O)O1)N(CC2=CN=C(C=C2)Cl)CC(F)F
Fluquinconazole	136426-54-5	Fc1ccc2N=C(n3cncn3)N(C(=O)c2c1)c4ccc(Cl)cc4Cl
Flurochloridon	61213-25-0	FC(F)(F)c1cccc(c1)N2CC(CCl)C(Cl)C2=O
Fluroxypyr	69377-81-7	Nc1c(Cl)c(F)nc(OCC(O)=O)c1Cl
Fluroxypyr-1-methylheptyl ester	81406-37-3	CCCCCCC(C)OC(=O)COc1nc(F)c(Cl)c(N)c1Cl
Flurprimidol	56425-91-3	CC(C)C(O)(c1ccc(OC(F)(F)F)cc1)c2cncnc2

Flusilazol	85509-19-9	C[Si](Cn1cncn1)(c2ccc(F)cc2)c3ccc(F)cc3
methyl 2-[2-chloro-4-fluoro-5-[(3-oxo-5,6,7,8-tetrahydro-[1,3,4]thiadiazolo[3,4-a]pyridazin-1-ylidene)amino]phenyl]sulfanylacetate	117337-19-6	COC(=O)CSc1cc(N=C2SC(=O)N3CCCCN23)c(F)cc1Cl
Flutianil	958647-10-4	COC1=CC=CC=C1N2CCSC2=C(C#N)SC3=C(C=CC(=C3)C(F)(F)F)F
Flutolanil	66332-96-5	CC(C)Oc1cccc(NC(=O)c2ccccc2C(F)(F)F)c1
Flutriafol	76674-21-0	OC(Cn1cncn1)(c2ccc(F)cc2)c3ccccc3F
Fluxapyroxad	907204-31-3	CN1C=C(C(=N1)C(F)F)C(=O)NC2=CC=CC=C2C3=CC(=C(C(=C3)F)F)F
Triflumizole metabolite FM-6-1	109849-99-2	CCCOCC(=NC1=C(C=C(C=C1)Cl)C(F)(F)F)N
Folpet	133-07-3	ClC(Cl)(Cl)SN1C(=O)c2ccccc2C1=O
Fonofos	944-22-9	CCO[P](=S)(CC)Sc1ccccc1
Forchlorfenuron	68157-60-8	Clc1cc(NC(=O)Nc2ccccc2)ccn1
Formetanate	22259-30-9	CNC(=O)Oc1cccc(c1)N=CN(C)C
Formetanate hydrochloride	23422-53-9	[H+].[Cl-].CNC(=O)Oc1cccc(c1)N=CN(C)C
Formothion	2540-82-1	CO[P](=S)(OC)SCC(=O)N(C)C=O
Fosthiazate	98886-44-3	CCO[P](=O)(SC(C)CC)N1CCSC1=O
Fuberidazol	3878-19-1	[nH]1c2ccccc2nc1c3occc3
Furalaxyl	57646-30-7	COC(=O)C(C)N(C(=O)c1occc1)c2c(C)cccc2C
Furathiocarb	65907-30-4	CCCCOC(=O)N(C)SN(C)C(=O)Oc1cccc2CC(C)(C)Oc12
Furmecyclox	60568-05-0	CON(C1CCCCC1)C(=O)c2cc(C)oc2C
Genite	97-16-5	Clc1ccc(O[S](=O)(=O)c2ccccc2)c(Cl)c1
Haloxyfop	69806-34-4	CC(Oc1ccc(Oc2ncc(cc2Cl)C(F)(F)F)cc1)C(O)=O
Haloxyfop-ethoxyethyl ester	87237-48-7	CCOCCOC(=O)C(C)OC1=CC=C(C=C1)OC2=C(C=C(C=N2)C(F)(F)F)Cl

Haloxyfop-methyl ester	69806-40-2	CC(C(=O)OC)OC1=CC=C(C=C1)OC2=C(C=C(C=N2)C(F)(F)F)Cl
HCH, alpha-	319-84-6	Cl[C@@H]1[C@H](Cl)[C@@H](Cl)[C@@H](Cl)[C@H](Cl)[C@H]1Cl
HCH, beta	319-84-6	Cl[C@H]1[C@H](Cl)[C@@H](Cl)[C@H](Cl)[C@@H](Cl)[C@@H]1Cl
HCH, delta-	319-86-8	Cl[C@@H]1[C@H](Cl)[C@@H](Cl)[C@H](Cl)[C@@H](Cl)[C@@H]1Cl
HCH, gamma- (Lindan)	58-89-9	Cl[C@@H]1[C@H](Cl)[C@@H](Cl)[C@H](Cl)[C@H](Cl)[C@H]1Cl
Heptachlor	76-44-8	ClC1C=CC2C1C3(Cl)C(=C(Cl)C2(Cl)C3(Cl)Cl)Cl
Heptachlorepoxid, cis- (exo,beta)	1024-57-3	Cl[C@H]1[C@H]2O[C@H]2[C@H]3[C@@H]1C4(Cl)C(Cl)=C(Cl)C3(Cl)C4(Cl)Cl
Heptachlorepoxid, trans	28044-83-9	[C@@H]12[C@@H]([C@H]([C@H]3[C@@H]1O3)Cl)[C@]4(C(=C([C@@]2(C4(Cl)Cl)Cl)Cl)Cl)Cl
Heptenophos	23560-59-0	CO[P](=O)(OC)OC1=C(Cl)C2C=CCC12
Hexachlorbenzol	118-74-1	Clc1c(Cl)c(Cl)c(Cl)c(Cl)c1Cl
Hexaconazol	79983-71-4	CCCCC(O)(Cn1cncn1)c2ccc(Cl)cc2Cl
Hexadecyltrimethylammonium chlorid	112-02-7	CCCCCCCCCCCCCCCC[N+](C)(C)C.[Cl-]
Hexadecyltrimethylammonium chlorid	112-02-7	CCCCCCCCCCCCCCCC[N+](C)(C)C.[Cl-]
Hexaflumuron	86479-06-3	FC(F)C(F)(F)Oc1c(Cl)cc(NC(=O)NC(=O)c2c(F)cccc2F)cc1Cl
Hexazinon	51235-04-2	CN1C(=NC(=O)N(C1=O)C2CCCCC2)N(C)C
Hexythiazox	78587-05-0	C[C@H]1C(SC(=O)N1C(=O)NC2CCCCC2)c3ccc(Cl)cc3
Hydramethylnon	67485-29-4	CC1(C)CNC(=NC1)N\N=C(/C=C/c2ccc(cc2)C(F)(F)F)\C=C\c3ccc(cc3)C(F)(F)F
Icaridin (Picaridin)	119515-38-7	CCC(C)OC(=O)N1CCCCC1CCO
Imazalil	35554-44-0	Clc1ccc(C(Cn2ccnc2)OCC=C)c(Cl)c1

Imazapyr	81334-34-1	CC(C)C1(C)N=C(NC1=O)c2ncccc2C(O)=O
Imazapyr	81334-34-1	CC(C)C1(C)N=C(NC1=O)c2ncccc2C(O)=O
Imazaquin	81335-37-7	CC(C)C1(C)N=C(NC1=O)c2nc3ccccc3cc2C(O)=O
Imazethapyr	81335-77-5	CCc1cnc(C2=NC(C)(C(C)C)C(=O)N2)c(c1)C(O)=O
Imibenconazole	86598-92-7	C1=CC(=CC=C1CSC(=NC2=C(C=C(C=C2)Cl)Cl)CN3C=NC=N3)Cl
Imidacloprid	138261-41-3	[O-][N+](=O)NC1=NCCN1Cc2ccc(Cl)nc2
Indoxacarb	173584-44-6	COC(=O)N(C(=O)N1CO[C@]2(Cc3cc(Cl)ccc3C2=N1)C(=O)OC)c4ccc(OC(F)(F)F)cc4
Iodofenphos	18181-70-9	CO[P](=S)(OC)Oc1cc(Cl)c(I)cc1Cl
Iodosulfuron-Methyl	144550-06-1	CC1=NC(=NC(=N1)OC)NC(=O)NS(=O)(=O)C2=C(C=CC(=C2)I)C(=O)OC
Ioxynil	1689-83-4	Oc1c(I)cc(cc1I)C#N
Ioxynil-Methyl	3336-40-1	CC1=NC(=NC(=N1)OC)NC(=O)NS(=O)(=O)C2=C(C=CC(=C2)I)C(=O)OC
Ioxynil Octanoate	3861-47-0	CCCCCCCC(=O)OC1=C(C=C(C=C1I)C#N)I
Iprobenfos	26087-47-8	CC(C)O[P](=O)(OC(C)C)SCc1ccccc1
Iprodione	36734-19-7	CC(C)NC(=O)N1CC(=O)N(C1=O)c2cc(Cl)cc(Cl)c2
Iprovalicarb	140923-17-7	CC(C)OC(=O)N[C@H](C(C)C)C(=O)NC(C)c1ccc(C)cc1
Isazofos	42509-80-8	CCO[P](=S)(OCC)Oc1nn(C(C)C)c(Cl)n1
Isobenzan	297-78-9	ClC1OC(Cl)C2C1C3(Cl)C(=C(Cl)C2(Cl)C3(Cl)Cl)Cl
Isocarbamid	30979-48-7	CC(C)CNC(=O)N1CCNC1=O
Isocarbophos	24353-61-5	CO[P](N)(=S)Oc1ccccc1C(=O)OC(C)C
Isodrine	465-73-6	[H][C@@]12C3CC(C=C3)[C@]1([H])[C@]4(Cl)C(Cl)=C(Cl)[C@@]2(Cl)C4(Cl)Cl
Isofenphos	25311-71-1	CCO[P](=S)(NC(C)C)Oc1ccccc1C(=O)OC(C)C
Isofenphos-Methyl	99675-03-3	CO[P](=S)(NC(C)C)Oc1ccccc1C(=O)OC(C)C

Isofenphosoxon	31120-85-1	CCO[P](=O)(NC(C)C)Oc1ccccc1C(=O)OC(C)C
Isofetamid	875915-78-9	CC1=C(SC=C1)C(=O)NC(C)(C)C(=O)C2=C(C=C(C=C2)OC(C)C)C
Isomethiozin	57052-04-7	CSC1=NN=C(C(=O)N1\N=C\C(C)C)C(C)(C)C
Isoprocarb	2631-40-5	CNC(=O)Oc1ccccc1C(C)C
Isopropalin	33820-53-0	CCCN(CCC)c1c(cc(cc1[N+]([O-])=O)C(C)C)[N+]([O-])=O
Isoprothiolane	50512-35-1	CC(C)OC(=O)C(C(=O)OC(C)C)=C1SCCS1
Isoproturon	34123-59-6	CC(C)c1ccc(NC(=O)N(C)C)cc1
Isopyrazam	881685-58-1	CC(C)C1C2CCC1C3=C2C=CC=C3NC(=O)C4=CN(N=C4C(F)F)C
Isoxaben	82558-50-7	CCC(C)(CC)c1cc(NC(=O)c2c(OC)cccc2OC)on1
Isoxadifen-Ethyl	163520-33-0	CCOC(=O)C1=NOC(C1)(c2ccccc2)c3ccccc3
Isoxaflutol	141112-29-0	CS(=O)(=O)C1=C(C=CC(=C1)C(F)(F)F)C(=O)C2=C(ON=C2)C3CC3
Isoxaflutole diketonitril	143701-75-1	CS(=O)(=O)C1=C(C=CC(=C1)C(F)(F)F)C(=O)C2=C(ON=C2)C3CC3
Isoxathion	18854-01-8	CCO[P](=S)(OCC)Oc1cc(on1)c2ccccc2
Jasmolin I	4466-14-2	CCC=CCC1=C(C(CC1=O)OC(=O)C2C(C2(C)C)C=C(C)C)C
Karanjin	521-88-0	COC1=C(Oc2c(ccc3occc23)C1=O)c4ccccc4
Kresoxim-methyl	143390-89-0	CO\N=C(C(=O)OC)/c1ccccc1COc2ccccc2C
Leptophos	21609-90-5	CO[P](=S)(Oc1cc(Cl)c(Br)cc1Cl)c2ccccc2
Linuron	330-55-2	CON(C)C(=O)Nc1ccc(Cl)c(Cl)c1
Lufenuron	103055-07-8	FC(C(F)(F)F)C(F)(F)Oc1cc(Cl)c(NC(=O)NC(=O)c2c(F)cccc2F)cc1Cl
Malaoxon	1634-78-2	CCOC(=O)CC(S[P](=O)(OC)OC)C(=O)OCC
Malathion	121-75-5	CCOC(=O)CC(S[P](=S)(OC)OC)C(=O)OCC
Mandestrobin	173662-97-0	CC1=CC(=C(C=C1)C)OCC2=CC=CC=C2C(C(=O)NC)OC

Mandipropamid	374726-62-2	COC1=C(C=CC(=C1)CCNC(=O)C(C2=CC=C(C=C2)Cl)OCC#C)OCC#C
Matrine	519-02-8	C1CC2C3CCCN4C3C(CCC4)CN2C(=O)C1
MCPA	94-74-6	Cc1cc(Cl)ccc1OCC(O)=O
MCPB	94-81-5	Cc1cc(Cl)ccc1OCCCC(O)=O
Mecarbam	2595-54-2	CCOC(=O)N(C)C(=O)CS[P](=S)(OCC)OCC
Mecoprop	93-65-2	CC(Oc1ccc(Cl)cc1C)C(O)=O
Mefenpyr-diethyl	135590-91-9	CCOC(=O)C1=NN(c2ccc(Cl)cc2Cl)C(C)(C1)C(=O)OCC
Mefentrifluconazole	1417782-03-6	CC(CN1C=NC=N1)(C2=C(C=C(C=C2)OC3=CC=C(C=C3)Cl)C(F)(F)F)O
Mepanipyrim	110235-47-7	CC#Cc1cc(C)nc(Nc2ccccc2)n1
Mephosfolan	950-10-7	CCO[P](=O)(OCC)N=C1SCC(C)S1
Mepronil	55814-41-0	CC(C)Oc1cccc(NC(=O)c2ccccc2C)c1
Merphos	150-50-5	CCCCSP(SCCCC)SCCCC
Metaflumizone	139968-49-3	C1=CC(=CC(=C1)C(F)(F)F)C(=NNC(=O)NC2=CC=C(C=C2)OC(F)(F)F)CC3=CC=C(C=C3)C#N
Metalaxyl	57837-19-1	COCC(=O)N(C(C)C(=O)OC)c1c(C)cccc1C
Metamitron	41394-05-2	CC1=NN=C(c2ccccc2)C(=O)N1N
Metazachlor	67129-08-2	Cc1cccc(C)c1N(Cn2cccn2)C(=O)CCl
Metazachlor ESA (479M08)	172960-62-2	CC1=C(C(=CC=C1)C)N(CN2C=CC=N2)C(=O)CS(=O)(=O)O
Metazachlor OA (479M04)	1231244-60-2	CC1=C(C(=CC=C1)C)N(CN2C=CC=N2)C(=O)C(=O)O
Metconazol	125116-23-6	CC1(CCC(C1(CN2C=NC=N2)O)CC3=CC=C(C=C3)Cl)C
Methabenzthiazuron	18691-97-9	CNC(=O)N(C)c1sc2ccccc2n1
Methacrifos	62610-77-9	COC(=O)C(/C)=C/O[P](=S)(OC)OC
Methamidophos	10265-92-6	CO[P](N)(=O)SC
Methidathion	950-37-8	COC1=NN(CS[P](=S)(OC)OC)C(=O)S1
Methiocarb	2032-65-7	CNC(=O)Oc1cc(C)c(SC)c(C)c1

Methiocarb-Sulfone	2179-25-1	CNC(=O)Oc1cc(C)c(c(C)c1)[S](C)(=O)=O
Methomyl	16752-77-5	CNC(=O)O\N=C(/C)SC
Methoprotryn	841-06-5	COCCCNc1nc(NC(C)C)nc(SC)n1
Methoxychlor	72-43-5	COc1ccc(cc1)C(c2ccc(OC)cc2)C(Cl)(Cl)Cl
Methoxyfenozide	161050-58-4	CC1=CC(=CC(=C1)C(=O)N(C(C)(C)C)NC(=O)C2=C(C(=CC=C2)OC)C)C
Metobromuron	3060-89-7	CON(C)C(=O)Nc1ccc(Br)cc1
Metolachlor	51218-45-2	CCc1cccc(C)c1N(C(C)COC)C(=O)CCl
Metolcarb	1129-41-5	CNC(=O)Oc1cccc(C)c1
Metominostrobin	133408-50-1	CNC(=O)C(=NOC)C1=CC=CC=C1OC2=CC=CC=C2
Metoxuron	19937-59-8	COc1ccc(NC(=O)N(C)C)cc1Cl
Metrafenone	220899-03-6	CC1=CC(=C(C(=C1C(=O)C2=C(C=CC(=C2C)Br)OC)OC)OC)OC
Metribuzin	21087-64-9	CSC1=NN=C(C(=O)N1N)C(C)(C)C
Metsulfuron-Methyl	74223-64-6	COC(=O)c1ccccc1[S](=O)(=O)NC(=O)Nc2nc(C)nc(OC)n2
Mevinphos (Summe der E- und Z-Isomeren)	298-01-1	CC(=CC(=O)OC)OP(=O)(OC)OC
Mirex	2385-85-5	ClC1(Cl)C2(Cl)C3(Cl)C4(Cl)C(Cl)(Cl)C5(Cl)C3(Cl)C1(Cl)C5(Cl)C24Cl
Molinat	2212-67-1	CCSC(=O)N1CCCCCC1
Monocrotophos	6923-22-4	CNC(=O)\C=C(/C)O[P](=O)(OC)OC
Monolinuron	1746-81-2	CON(C)C(=O)Nc1ccc(Cl)cc1
Monuron	150-68-5	CN(C)C(=O)Nc1ccc(Cl)cc1
Myclobutanil	88671-89-0	CCCCC(Cn1cncn1)(C#N)c2ccc(Cl)cc2
N-2.4-Dimethylphenyl-N'-methylformamidine [DMPF]	33089-74-6	CC1=CC(=C(C=C1)NC=NC)C
Naled	300-76-5	CO[P](=O)(OC)OC(Br)C(Cl)(Cl)Br
Naphtyloxyacetamid	35368-77-5	C1=CC=C2C=C(C=CC2=C1)OCC(=O)N
Napropamid	15299-99-7	CCN(CC)C(=O)C(C)Oc1cccc2ccccc12
Neburon	555-37-3	CCCCN(C)C(=O)Nc1ccc(Cl)c(Cl)c1

Nicosulfuron	111991-09-4	COc1cc(OC)nc(NC(=O)N[S](=O)(=O)c2ncccc2C(=O)N(C)C)n1
Nicotin	54-11-5	CN1CCCC1c2cccnc2
Nitenpyram	150824-47-8	CCN(Cc1ccc(Cl)nc1)\C(NC)=C\[N+]([O-])=O
Nitrapyrin	1929-82-4	Clc1cccc(n1)C(Cl)(Cl)Cl
Nitrofen	1836-75-5	[O-][N+](=O)c1ccc(Oc2ccc(Cl)cc2Cl)cc1
Nitrothal-Isopropyl	10552-74-6	CC(C)OC(=O)c1cc(cc(c1)[N+]([O-])=O)C(=O)OC(C)C
Norflurazon	27314-13-2	CNC1=C(Cl)C(=O)N(N=C1)c2cccc(c2)C(F)(F)F
Novaluron	116714-46-6	FC(OC(F)(F)F)C(F)(F)Oc1ccc(NC(=O)NC(=O)c2c(F)cccc2F)cc1Cl
Nuarimol	63284-71-9	OC(c1ccc(F)cc1)(c2cncnc2)c3ccccc3Cl
Ofurac	58810-48-3	CC1=C(C(=CC=C1)C)N(C2CCOC2=O)C(=O)CCl
Omethoat	1113-02-6	CNC(=O)CS[P](=O)(OC)OC
Oxadiargyl	39807-15-3	CC(C)(C)C1=NN(C(=O)O1)c2cc(OCC#C)c(Cl)cc2Cl
Oxadiazon	19666-30-9	CC(C)Oc1cc(N2N=C(OC2=O)C(C)(C)C)c(Cl)cc1Cl
Oxadixyl	77732-09-3	COCC(=O)N(N1CCOC1=O)c2c(C)cccc2C
Oxamyl	23135-22-0	CNC(=O)O\N=C(SC)\C(=O)N(C)C
Oxathiapiprolin	1003318-67-9	CC1=CC(=NN1CC(=O)N2CCC(CC2)C3=NC(=CS3)C4=NOC(C4)C5=C(C=CC=C5F)F)C(F)(F)F
Oxycarboxin	5259-88-1	CC1=C(C(=O)Nc2ccccc2)[S](=O)(=O)CCO1
Oxydemeton-Methyl	301-12-2	CC[S](=O)CCS[P](=O)(OC)OC
Oxyfluorfen	42874-03-3	CCOc1cc(Oc2ccc(cc2Cl)C(F)(F)F)ccc1[N+]([O-])=O
Oxymatrine	16837-52-8	C1CC2C3CCC[N+]4(C3C(CCC4)CN2C(=O)C1)[O-]
Paclobutrazol	76738-62-0	CC(C)(C)C(C(CC1=CC=C(C=C1)Cl)N2C=NC=N2)O
Paraoxon	311-45-5	CCO[P](=O)(OCC)Oc1ccc(cc1)[N+]([O-])=O
Paraoxon-Methyl	950-35-6	CO[P](=O)(OC)Oc1ccc(cc1)[N+]([O-])=O

Parathion	56-38-2	CCO[P](=S)(OCC)Oc1ccc(cc1)[N+]([O-])=O
Parathion-Methyl	298-00-0	CO[P](=S)(OC)Oc1ccc(cc1)[N+]([O-])=O
Pebulat	1114-71-2	CCCCN(CC)C(=O)SCCC
Penconazol	66246-88-6	CCCC(Cn1cncn1)c2ccc(Cl)cc2Cl
Pencycuron	66063-05-6	Clc1ccc(CN(C2CCCC2)C(=O)Nc3ccccc3)cc1
Pencycuron-PB-amin	66063-15-8	C1CCC(C1)NCC2=CC=C(C=C2)Cl
Pendimethalin	40487-42-1	CCC(CC)Nc1c(cc(C)c(C)c1[N+]([O-])=O)[N+]([O-])=O
Penflufen	494793-67-8	CC1=NN(C(=C1C(=O)NC2=CC=CC=C2C(C)CC(C)C)F)C
Pentachloroaniline	527-20-8	C1(=C(C(=C(C(=C1Cl)Cl)Cl)Cl)Cl)N
Pentachloroanisole	1825-21-4	COC1=C(C(=C(C(=C1Cl)Cl)Cl)Cl)Cl
Pentachlorbenzol	608-93-5	Clc1cc(Cl)c(Cl)c(Cl)c1Cl
Pentachlorphenol	87-86-5	Oc1c(Cl)c(Cl)c(Cl)c(Cl)c1Cl
Pentanochlor	2307-68-8	CCCC(C)C(=O)Nc1ccc(C)c(Cl)c1
Penthiopyrad	183675-82-3	CC(C)CC(C)C1=C(C=CS1)NC(=O)C2=CN(N=C2C(F)(F)F)C
Permethrin	52645-53-1	CC1(C)[C@@H](C=C(Cl)Cl)[C@H]1C(=O)OCc2cccc(Oc3ccccc3)c2
Perthan	72-56-0	CCc1ccc(cc1)C(C(Cl)Cl)c2ccc(CC)cc2
Pethoxamid	106700-29-2	CCOCCN(C(=O)CCl)C(=C(C)C)c1ccccc1
Phenmedipham	13684-63-4	COC(=O)Nc1cccc(OC(=O)Nc2cccc(C)c2)c1
Phenothrin	26002-80-2	CC(C)=CC1C(C(=O)OCc2cccc(Oc3ccccc3)c2)C1(C)C
Phenylphenol, ortho-	90-43-7	Oc1ccccc1c2ccccc2
Phorate	298-02-2	CCO[P](=S)(OCC)SCSCC
Phorat-Oxon	2600-69-3	CCOP(=O)(OCC)SCSCC
Zolone	2310-17-0	CCO[P](=S)(OCC)SCN1C(=O)Oc2cc(Cl)ccc12
Phosfolan	947-02-4	CCO[P](=O)(OCC)N=C1SCCS1
Phosmet	732-11-6	CO[P](=S)(OC)SCN1C(=O)c2ccccc2C1=O
Phosmet Oxon	3735-33-9	CO[P](=O)(OC)SCN1C(=O)c2ccccc2C1=O

Phosphamidon	23783-98-4	CCN(CC)C(=O)\C(Cl)=C(/C)O[P](=O)(OC)OC
Phoxim	14816-18-3	CCO[P](=S)(OCC)O\N=C(C#N)\c1ccccc1
Phthalimide	85-41-6	O=C1NC(=O)c2ccccc12
Picolinafen	137641-05-5	Fc1ccc(NC(=O)c2cccc(Oc3cccc(c3)C(F)(F)F)n2)cc1
Picoxystrobin	117428-22-5	CO/C=C(C(=O)OC)/c1ccccc1COc2cccc(n2)C(F)(F)F
Piperonylbutoxide	51-03-6	CCCCOCCOCCOCC1=CC2=C(C=C1CCC)OCO2
Piperophos	24151-93-7	CCCO[P](=S)(OCCC)SCC(=O)N1CCCCC1C
Pirimicarb	23103-98-2	CN(C)C(=O)Oc1nc(nc(C)c1C)N(C)C
Pirimicarb, Desmethyl-	30614-22-3	CC1=C(N=C(N=C1OC(=O)N(C)C)NC)C
Pirimicarb	27218-04-8	CN(C)C(=O)Oc1nc(nc(C)c1C)N(C)C=O
Pirimiphos-Ethyl	23505-41-1	CCO[P](=S)(OCC)Oc1cc(C)nc(n1)N(CC)CC
Pirimiphos-Methyl	29232-93-7	CCN(CC)c1nc(C)cc(O[P](=S)(OC)OC)n1
Plifenat	21757-82-4	CC(=O)OC(C1=CC(=C(C=C1)Cl)Cl)C(Cl)(Cl)Cl
Prallethrin	23031-36-9	CC(C)=C[C@H]1[C@H](C(=O)OC2CC(=O)C(=C2C)CC#C)C1(C)C
Prochloraz	67747-09-5	CCCN(CCOc1c(Cl)cc(Cl)cc1Cl)C(=O)n2ccnc2
Prochloraz Metabolite BTS 44595	139520-94-8	ClC1=C(OCCN(C(N)=O)CCC)C(Cl)=CC(Cl)=C1
Prochloraz Metabolite BTS 44596	139520-94-8	ClC1=C(OCCN(CCC)C(NC([H])=O)=O)C(Cl)=CC(Cl)=C1
Procymidon	32809-16-8	CC12CC1(C)C(=O)N(C2=O)c3cc(Cl)cc(Cl)c3
Profenofos	41198-08-7	CCCS[P](=O)(OCC)Oc1ccc(Br)cc1Cl
Profluralin	26399-36-0	CCCN(CC1CC1)c2c(cc(cc2[N+]([O-])=O)C(F)(F)F)[N+]([O-])=O
2-[(E)-N-[2-(4-chlorophenoxy)propoxy]-C-propylcarbonimidoyl]-3-hydroxy-5-(thian-3-yl)cyclohex-2-en-1-one	139001-49-3	CCC\C(=N/OCC(C)Oc1ccc(Cl)cc1)C2=C(O)CC(CC2=O)C3CCCSC3
Promecarb	2631-37-0	CNC(=O)Oc1cc(C)cc(c1)C(C)C

Prometon	1610-18-0	COc1nc(NC(C)C)nc(NC(C)C)n1
Prometryn	7287-19-6	CSc1nc(NC(C)C)nc(NC(C)C)n1
Propachlor	1918-16-7	CC(C)N(C(=O)CCl)c1ccccc1
Propamocarb	24579-73-5	CCCOC(=O)NCCCN(C)C
Propanil	709-98-8	CCC(=O)Nc1ccc(Cl)c(Cl)c1
Propaquizafop	111479-05-1	CC(Oc1ccc(Oc2cnc3cc(Cl)ccc3n2)cc1)C(=O)OCCON=C(C)C
Propargit	2312-35-8	CC(C)(C)C1=CC=C(C=C1)OC2CCCCC2OS(=O)OCC#C
Propazin	139-40-2	CC(C)Nc1nc(Cl)nc(NC(C)C)n1
Propetamphos	31218-83-4	CCN[P](=S)(OC)OC(/C)=C\C(=O)OC(C)C
Propham	122-42-9	CC(C)OC(=O)Nc1ccccc1
Propiconazol	60207-90-1	CCCC1COC(O1)(CN2C=NC=N2)C3=C(C=C(C=C3)Cl)Cl
Propoxur	114-26-1	CNC(=O)Oc1ccccc1OC(C)C
Propylen Thiourea	2122-19-2	CC1CNC(=S)N1
Propyzamid	23950-58-5	CC(C)(C#C)NC(=O)C1=CC(=CC(=C1)Cl)Cl
Proquinazid	189278-12-4	CCCN1C(=O)C2=C(C=CC(=C2)I)N=C1OCCC
Prosulfocarb	52888-80-9	CCCN(CCC)C(=O)SCc1ccccc1
Prosulfuron	94125-34-5	COc1nc(C)nc(NC(=O)N[S](=O)(=O)c2ccccc2CCC(F)(F)F)n1
Prothioconazol	178928-70-6	C1CC1(C(CC2=CC=CC=C2Cl)(CN3C(=S)N=CN3)O)Cl
Prothioconazol-desthio	120983-64-4	OC(Cc1ccccc1Cl)(Cn2cncn2)C3(Cl)CC3
Prothiofos	34643-46-4	CCCS[P](=S)(OCC)Oc1ccc(Cl)cc1Cl
Pymetrozin	123312-89-0	CC1=NNC(=O)N(C1)\N=C\c2cccnc2
Pyraclostrobin	175013-18-0	CON(C(=O)OC)c1ccccc1COc2ccn(n2)c3ccc(Cl)cc3
Pyraflufen	129630-17-7	CN1C(=C(C(=N1)C2=CC(=C(C=C2F)Cl)OCC(=O)O)Cl)OC(F)F
Pyraflufen-Ethyl	129630-19-9	CCOC(=O)COc1cc(c(F)cc1Cl)c2nn(C)c(OC(F)F)c2Cl
Pyrazophos	13457-18-6	CCOC(=O)c1cn2nc(O[P](=S)(OCC)OCC)cc2nc1C

Pyrethrin I	121-29-9	CC1=C(C(=O)CC1OC(=O)C2C(C2(C)C)C=C(C)C)CC=CC=C
Pyridaben	96489-71-3	CC(C)(C)N1N=CC(=C(Cl)C1=O)SCc2ccc(cc2)C(C)(C)C
Pyridafol	40020-01-7	C1=CC=C(C=C1)C2=NNC(=CC2=O)Cl
Pyridafol	40020-01-7	C1=CC=C(C=C1)C2=NNC(=CC2=O)Cl
Pyridalyl	179101-81-6	FC(F)(F)c1ccc(OCCCOc2c(Cl)cc(OCC=C(Cl)Cl)cc2Cl)nc1
Pyridaphenthion	119-12-0	CCO[P](=S)(OCC)OC1=NN(C(=O)C=C1)c2ccccc2
Pyridat	55512-33-9	CCCCCCCCSC(=O)Oc1cc(Cl)nnc1c2ccccc2
Pyrifenox	88283-41-4	CO\N=C(\Cc1cccnc1)c2ccc(Cl)cc2Cl
Pyrimethanil	53112-28-0	Cc1cc(C)nc(Nc2ccccc2)n1
Pyriofenon	688046-61-9	CC1=CC(=C(C(=C1C(=O)C2=C(C(=CN=C2OC)Cl)C)OC)OC)OC
Pyriproxyfen	121-21-1	CC(COc1ccc(Oc2ccccc2)cc1)Oc3ccccn3
Quinalphos	13593-03-8	CCO[P](=S)(OCC)Oc1cnc2ccccc2n1
Quinclorac	84087-01-4	OC(=O)c1c(Cl)ccc2cc(Cl)cnc12
Quinmerac	90717-03-6	Cc1cnc2c(ccc(Cl)c2C(O)=O)c1
quinoclamine	2797-51-5	NC1=C(Cl)C(=O)c2ccccc2C1=O
Quinoxyfen	124495-18-7	Fc1ccc(Oc2ccnc3cc(Cl)cc(Cl)c23)cc1
Quintozen	82-68-8	[O-][N+](=O)c1c(Cl)c(Cl)c(Cl)c(Cl)c1Cl
Quizalofop-Ethyl	76578-14-8	CCOC(=O)C(C)Oc1ccc(Oc2cnc3cc(Cl)ccc3n2)cc1
Quizalofop-P	94051-08-8	C[C@@H](Oc1ccc(Oc2cnc3cc(Cl)ccc3n2)cc1)C(O)=O
Rabenzazol	40341-04-6	Cc1cc(C)n(n1)c2[nH]c3ccccc3n2
Resmethrin	10453-86-8	CC(C)=CC1C(C(=O)OCc2coc(Cc3ccccc3)c2)C1(C)C
Rimsulfuron	122931-48-0	CC[S](=O)(=O)c1cccnc1[S](=O)(=O)NC(=O)Nc2nc(OC)cc(OC)n2

Rotenon	83-79-4	COc1cc2OC[C@H]3Oc4c5C[C@@H](Oc5ccc4C(=O)[C@H]3c2cc1OC)C(C)=C
Chlorantraniliprole	500008-45-7	CC1=CC(=CC(=C1NC(=O)C2=CC(=NN2C3=C(C=CC=N3)Cl)Br)C(=O)NC)Cl
S421	127-90-2	ClC(COCC(Cl)C(Cl)(Cl)Cl)C(Cl)(Cl)Cl
Saflufenacil	372137-35-4	CC(C)N(C)S(=O)(=O)NC(=O)C1=CC(=C(C=C1Cl)F)N2C(=O)C=C(N(C2=O)C)C(F)(F)F
Saflufenacil M800H11	1246768-30-8	O=C(C=C(C(F)(F)F)N1)N(C2=CC(C(NS(NC(C)C)(=O)=O)=O)=C(Cl)C=C2F)C1=O
Saflufenacil M800H35	1246768-31-9	O=C(NS(NC(C)C)(=O)=O)C1=C(Cl)C=C(F)C(NC(N)=O)=C1
Salflufenacil Metabolite M800H35	1246768-31-9	O=C(NS(NC(C)C)(=O)=O)C1=C(Cl)C=C(F)C(NC(N)=O)=C1
Saflufenacil Metabolite M800H11	1246768-30-8	O=C(C=C(C(F)(F)F)N1)N(C2=CC(C(NS(NC(C)C)(=O)=O)=O)=C(Cl)C=C2F)C1=O
Sebuthylazin	7286-69-3	CCNc1nc(Cl)nc(NC(C)CC)n1
Sethoxydim	74051-80-2	CCCC(=NOCC)C1=C(CC(CC1=O)CC(C)SCC)O
Silafluofen	105024-66-6	CCOc1ccc(cc1)[Si](C)(C)CCCc2ccc(F)c(Oc3ccccc3)c2
Silthiofam	175217-20-6	CC1=C(SC(=C1C(=O)NCC=C)[Si](C)(C)C)C
Simazine	122-34-9	CCNc1nc(Cl)nc(NCC)n1
Sintofen	130561-48-7	COCCOc1cccc2N(N=C(C(O)=O)C(=O)c12)c3ccc(C)cc3
Solatenol	1072957-71-1	CN1C=C(C(=N1)C(F)F)C(=O)NC2=CC=CC3=C2C4CCC3C4=C(Cl)Cl
Spinetoram J	187166-40-1	CCC1CCCC(C(C(=O)C2=CC3C(C2CC(=O)O1)CCC4C3CC(C4)OC5C(C(C(C(O5)C)OC)OCC)OC)C)OC6CCC(C(O6)C)N(C)C
Spinosyn A	131929-60-7	CCC1CCCC(C(C(=O)C2=CC3C4CC(CC4C=CC3C2CC(=O)O1)OC5C(C(C(C(O5)C)OC)OC)OC)C)OC6CCC(C(O6)C)N(C)C
Spirodiclofen	148477-71-8	CCC(C)(C)C(=O)OC1=C(C(=O)OC12CCCCC2)c3ccc(Cl)cc3Cl

Spiromesifen	283594-90-1	Cc1cc(C)c(c(C)c1)C2=C(OC(=O)CC(C)(C)C)C3(CCCC3)OC2=O
Spirotetramat	203313-25-1	CCOC(=O)OC1=C(C(=O)NC12CCC(CC2)OC)C3=C(C=CC(=C3)C)C
Spirotetramat-enol	382608-09-5	CC1=CC(=C(C=C1)C)C2C(=O)C3(CCC(CC3)OC)NC2=O
Spirotetramat-enol-glucoside	1172614-86-6	COC1CCC2(CC1)NC(=O)C(=C2O[C@@H]3O[C@H](CO)[C@@H](O)[C@H](O)[C@H]3O)c4cc(C)ccc4C
Spirotetramat keto hydroxy	1172134-11-0	COC1CCC2(CC1)NC(=O)C(O)(C2=O)c3cc(C)ccc3C
Spirotetramat mono hydroxy	1172134-12-1	COC1CCC2(CC1)NC(=O)C(C2O)c3cc(C)ccc3C
Spiroxamine	118134-30-8	CCCN(CC)CC1COC2(CCC(CC2)C(C)(C)C)O1
Sulcotrione	99105-77-8	C[S](=O)(=O)c1ccc(c(Cl)c1)C(=O)C2C(=O)CCCC2=O
Sulfentrazone	122836-35-5	CC1=NN(C(=O)N1C(F)F)c2cc(N[S](C)(=O)=O)c(Cl)cc2Cl
Sulfosulfuron	141776-32-1	CC[S](=O)(=O)c1nc2ccccn2c1[S](=O)(=O)NC(=O)Nc3nc(OC)cc(OC)n3
Sulfotep	3689-24-5	CCO[P](=S)(OCC)O[P](=S)(OCC)OCC
Sulfoxaflor	946578-00-3	CC(C1=CN=C(C=C1)C(F)(F)F)S(=NC#N)(=O)C
Sulprofos	35400-43-2	CCCS[P](=S)(OCC)Oc1ccc(SC)cc1
Swep	1918-18-9	COC(=O)Nc1ccc(Cl)c(Cl)c1
Klartan	102851-06-9	CC(C)C(Nc1ccc(cc1Cl)C(F)(F)F)C(=O)OC(C#N)c2cccc(Oc3ccccc3)c2
Tebuconazol	107534-96-3	CC(C)(C)C(CCC1=CC=C(C=C1)Cl)(CN2C=NC=N2)O
Tebufenozide	112410-23-8	CCc1ccc(cc1)C(=O)NN(C(=O)c2cc(C)cc(C)c2)C(C)(C)C
Tebufenpyrad	119168-77-3	CCc1nn(C)c(C(=O)NCc2ccc(cc2)C(C)(C)C)c1Cl
Tebupirimphos	96182-53-5	CCO[P](=S)(OC(C)C)Oc1cnc(nc1)C(C)(C)C
Tebutam	35256-85-0	CC(C)N(Cc1ccccc1)C(=O)C(C)(C)C
Tecnazen	117-18-0	[O-][N+](=O)c1c(Cl)c(Cl)cc(Cl)c1Cl

Teflubenzuron	83121-18-0	Fc1cccc(F)c1C(=O)NC(=O)Nc2cc(Cl)c(F)c(Cl)c2F
Tefluthrin	79538-32-2	Cc1c(F)c(F)c(COC(=O)C2C(\C=C(/Cl)C(F)(F)F)C2(C)C)c(F)c1F
Tembotrione	335104-84-2	CS(=O)(=O)C1=C(C(=C(C=C1)C(=O)C2C(=O)CCCC2=O)Cl)COCC(F)(F)F
Temephos	3383-96-8	CO[P](=S)(OC)Oc1ccc(Sc2ccc(O[P](=S)(OC)OC)cc2)cc1
Tepraloxydim	149979-41-9	CCC(=NOCC=CCl)C1=C(CC(CC1=O)C2CCOCC2)O
Terbacil	5902-51-2	CC1=C(Cl)C(=O)N(C(=O)N1)C(C)(C)C
Terbufos	13071-79-9	CCO[P](=S)(OCC)SCSC(C)(C)C
Terbufos-sulfone	56070-16-7	CCO[P](=S)(OCC)SC[S](=O)(=O)C(C)(C)C
Terbumeton	33693-04-8	CCNc1nc(NC(C)(C)C)nc(OC)n1
Terbuthylazin	5915-41-3	CCNc1nc(Cl)nc(NC(C)(C)C)n1
Terbutryn	886-50-0	CCNc1nc(NC(C)(C)C)nc(SC)n1
Tetrachlorvinphos	22248-79-9	CO[P](=O)(OC)OC(=C/Cl)/c1cc(Cl)c(Cl)cc1Cl
Tetraconazol	112281-77-3	C1=CC(=C(C=C1Cl)Cl)C(CN2C=NC=N2)COC(C(F)F)(F)F
Tetradifon	116-29-0	Clc1ccc(cc1)[S](=O)(=O)c2cc(Cl)c(Cl)cc2Cl
Tetramethrin	7696-12-0	CC(C)=C[C@@H]1[C@@H](C(=O)OCN2C(=O)C3=C(CCCC3)C2=O)C1(C)C
TFNA	158063-66-2	C1=CN=CC(=C1C(F)(F)F)C(=O)O
TFNG	207502-65-6	C1=CN=CC(=C1C(F)(F)F)C(=O)NCC(=O)O
Thiabendazol	148-79-8	[nH]1c2ccccc2nc1c3cscn3
5-Hydroxythiabendazole	948-71-0	Oc1ccc2nc([nH]c2c1)c3cscn3
Thiacloprid	111988-49-9	Clc1ccc(CN2CCSC2=NC#N)cn1
Thiamethoxam	153719-23-4	CN1COCN(Cc2sc(Cl)nc2)\C1=N/[N+]([O-])=O
Thifensulfuron methyl	79277-27-3	COC(=O)c1sccc1[S](=O)(=O)NC(=O)Nc2nc(C)nc(OC)n2
Thiodicarb	59669-26-0	CSC(\C)=N/OC(=O)N(C)SN(C)C(=O)O\N=C(\C)SC

Thiofanox	39196-18-4	CNC(=O)O\N=C(/CSC)C(C)(C)C
Thiofanox-sulfon	39184-59-3	CNC(=O)O/N=C(/C[S](C)(=O)=O)C(C)(C)C
Thiofanox-sulfoxide	39184-27-5	CNC(=O)O\N=C(\C[S](C)=O)C(C)(C)C
Thiometon	640-15-3	CCSCCS[P](=S)(OC)OC
Thionazin	297-97-2	CCO[P](=S)(OCC)Oc1cnccn1
Methylthiofanate	23564-05-8	COC(=O)NC(=S)Nc1ccccc1NC(=S)NC(=O)OC
Tiocarbazil	36756-79-3	CCC(C)N(C(C)CC)C(=O)SCc1ccccc1
Tolclofos-Methyl	57018-04-9	CO[P](=S)(OC)Oc1c(Cl)cc(C)cc1Cl
Tolfenpyrad	129558-76-5	CCc1nn(C)c(C(=O)NCc2ccc(Oc3ccc(C)cc3)cc2)c1Cl
Tolylfluanid	731-27-1	CN(C)[S](=O)(=O)N(SC(F)(Cl)Cl)c1ccc(C)cc1
Tralkoxydim	87820-88-0	CCC(=NOCC)C1=C(CC(CC1=O)C2=C(C=C(C=C2C)C)C)O
Transfluthrin	118712-89-3	CC1(C)[C@H](C=C(Cl)Cl)[C@H]1C(=O)OCc2c(F)c(F)cc(F)c2F
Triadimefon	43121-43-3	CC(C)(C)C(=O)C(Oc1ccc(Cl)cc1)n2cncn2
Triadimenol	55219-65-3	CC(C)(C)C(O)C(Oc1ccc(Cl)cc1)n2cncn2
Triallat	2303-17-5	CC(C)N(C(C)C)C(=O)SCC(=C(Cl)Cl)Cl
Triasulfuron	82097-50-5	COc1nc(C)nc(NC(=O)N[S](=O)(=O)c2ccccc2OCCCl)n1
Triazophos	24017-47-8	CCO[P](=S)(OCC)Oc1ncn(n1)c2ccccc2
Tribenuron methyl	101200-48-0	COC(=O)c1ccccc1[S](=O)(=O)NC(=O)N(C)c2nc(C)nc(OC)n2
Trichlorfon	52-68-6	CO[P](=O)(OC)C(O)C(Cl)(Cl)Cl
Trichloronat	327-98-0	CCO[P](=S)(CC)Oc1cc(Cl)c(Cl)cc1Cl
Tricyclazol	41814-78-2	Cc1cccc2sc3nncn3c12
Tridemorph	24602-86-6	CCCCCCCCCCCCCN1CC(C)OC(C)C1
Tridiphane	58138-08-2	Clc1cc(Cl)cc(c1)C2(CO2)CC(Cl)(Cl)Cl
Trietazine	1912-26-1	CCNc1nc(Cl)nc(n1)N(CC)CC

Trifloxystrobin	141517-21-7	CO\N=C(C(=O)OC)/c1ccccc1CO\N=C(/C)c2cccc(c2)C(F)(F)F
Triflumizole	68694-11-1	CCCOCC(=Nc1ccc(Cl)cc1C(F)(F)F)n2ccnc2
Triflumuron	64628-44-0	FC(F)(F)Oc1ccc(NC(=O)NC(=O)c2ccccc2Cl)cc1
Trifluralin	1582-09-8	CCCN(CCC)c1c(cc(cc1[N+]([O-])=O)C(F)(F)F)[N+]([O-])=O
Upbeet	126535-15-7	COC(=O)c1cccc(C)c1[S](=O)(=O)NC(=O)Nc2nc(OCC(F)(F)F)nc(n2)N(C)C
Triforin	26644-46-2	ClC(Cl)(Cl)C(NC=O)N1CCN(CC1)C(NC=O)C(Cl)(Cl)Cl
Trinexapac	143294-89-7	C1CC1C(=C2C(=O)CC(CC2=O)C(=O)O)O
Triticonazole	131983-72-7	CC1(C)CC\C(=C/c2ccc(Cl)cc2)C1(O)Cn3cncn3
Tritosulfuron	142469-14-5	COc1nc(NC(=O)N[S](=O)(=O)c2ccccc2C(F)(F)F)nc(n1)C(F)(F)F
Uniconazole	83657-22-1	CC(C)(C)C(O)\C(=C/c1ccc(Cl)cc1)n2cncn2
Valifenalate	283159-90-0	CC(C)C(C(=O)NC(CC(=O)OC)C1=CC=C(C=C1)Cl)NC(=O)OC(C)C
Vamidothion	2275-23-2	CNC(=O)C(C)SCCS[P](=O)(OC)OC
Vinclozolin	50471-44-8	CC1(OC(=O)N(C1=O)c2cc(Cl)cc(Cl)c2)C=C
XMC	2655-14-3	CNC(=O)Oc1cc(C)cc(C)c1
Zoxamid	156052-68-5	CCC(C)(C(=O)CCl)NC(=O)C1=CC(=C(C(=C1)Cl)C)Cl

Table S2. Detection results for solution "A" containing 372 pesticides across different ionization schemes and concentrations. Each reagent ion (Br^-, H_3O^+, O_2^-, and protonated acetone) was tested at varying target concentrations and injection volumes, with the number and percentage of detected targets recorded.

Solution "A" containing 372 pesticides

**Reagent ion precursors**		**Concentration per target (pg/µl)**	**Injection volume (µl)**	**Number of detected targets**	**Percentage of detected targets (%)**
		10	10	83	18
		20	10	123	29
Br^-	Br adduct	100	10	166	43
		1000	1	174	44
		2500	1	187	46
		10	10	123	30
		20	10	236	58
H_3O^+	Protonated	100	10	272	67
		1000	1	274	68
		2500	1	281	70
		10	10	35	9
		20	10	59	15
O_2^-	Deprotonated	100	10	91	23
		1000	1	124	31
		2500	1	127	31
		10	10	158	39
		20	10	215	53
$(C_3H_6O + H)^+$	Protonated	100	10	262	65
		1000	1	287	71
		2500	1	288	71

Table S3. Detection results for solution 'B' containing 279 pesticides using various ionization schemes and concentrations. The table shows the number and percentage of detected targets for each reagent ion (Br^-, H_3O^+, O_2^-, and protonated acetone) at specified concentrations and injection volumes.

Solution "B" containing 279 pesticides

**Reagent ion precursors**		**Concentration per target (pg/µl)**	**Injection volume (µl)**	**Number of detected targets**	**Percentage of detected targets (%)**
Br^-	Br adduct	10	10	37	12
		20	10	58	19
		100	10	91	29
		1000	1	86	28
		2500	1	120	38
H_3O^+	Protonated	10	10	114	37
		20	10	158	51
		100	10	194	62
		1000	1	193	62
		2500	1	199	64
O_2^-	Deprotonated	10	10	21	7
		20	10	34	11
		100	10	60	19
		1000	1	83	27
		2500	1	97	31
$(C_3H_6O + H)^+$	Protonated	10	10	128	41
		20	10	178	57
		100	10	161	52
		1000	1	187	60
		2500	1	197	63

Table S4. Pesticides detected in each fruit and vegetable extract as measured by Finnish Customs using validated GC-MS/MS and UHPLC-MS/MS methods.

Extract	Pesticide	Result (mg/kg)
Mandarin	imazalil	0.83
Bell paper	acetamiprid	0.01
	imidacloprid	0.021
	azoxystrobin	0.011
Tomato	Fluopyram	0.021
Pomelo	Acetamiprid	0.011
	Cypermethrin	0.074
Pineapple A	Fludioxonil	0.48
	Diazinon	0.011
Lemon	Carbendazim	0.074
	Pyrimethanil	0.89
	thiabendazol	0.81
	Imazalil	1
	Pyraclostrobin	0.027
	Pyriproxyfen	0.015
Grapefruit	Methoxyfenozide	0.018
	sulfoxaflor	0.014
	Imazalil	0.51
	Pyraclostrobin	0.018
Orange A	pyrimethanil	1.5
	thiabendazol	1.5
	imazalil	2.1
	trifloxystrobin	0.026
Orange B	pyrimethanil	2.3
	thiabendazol	1.1
	imazalil	1.8
	trifloxystrobin	0.015
	propiconazol	0.012
	fenpyroximat	0.023
Persimmon	sulfoxaflor	0.014
	etofenprox	0.045
Rucola	mandipropamid	0.3
	alpha-cypermethrin	0.023
	cypermethrin	0.023
Spinach	boscalid	2.4
	pyraclostrobin	0.22
Pear		
Strawberry	boscalid	0.22
	ethirimol	0.016
	fluopyram	0.2
	Bupirimat	0.12
	pyraclostrobin	0.034
	trifloxystrobin	0.16

	clofentezin	0.023
	spinosad	0.079
	cyprodinil	0.48
	penconazol	0.022
	Fludioxonil	0.4
Pinaapple B	Fludioxonil	0.3
Paprika	Pyridalyl	0.061
	Flonicamid	0.024
	TFNA	0.011
	TFNG	0.14
	Dicofol-p,p	0.012
	Azoxystrobin	0.011

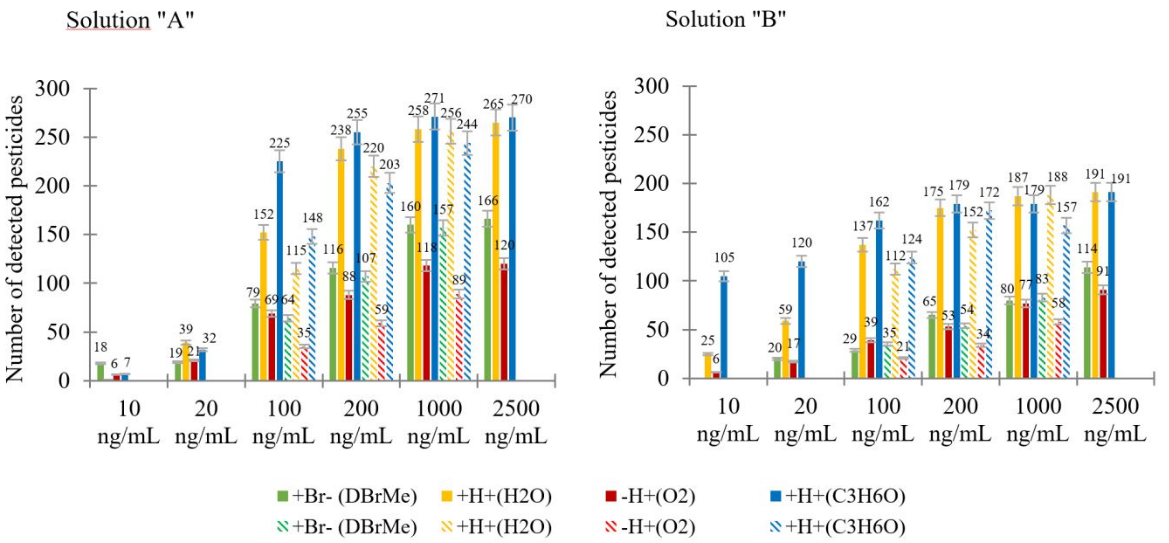

Figure S1. The effect of increasing sample loading on the number of detected pesticides across all ionization schemes for solutions “A” and “B”. While increasing the injected amount initially improves detection, a saturation trend is observed beyond approximately 1 ng, with no significant gain in the number of detected pesticides. This indicates non-linear detection scaling and suggests that higher loading does not necessarily enhance coverage. Error bars represent measurement variability.

Figure S2. Green diagram is showing the favorable channel for protonation and has the least energy.

(a)

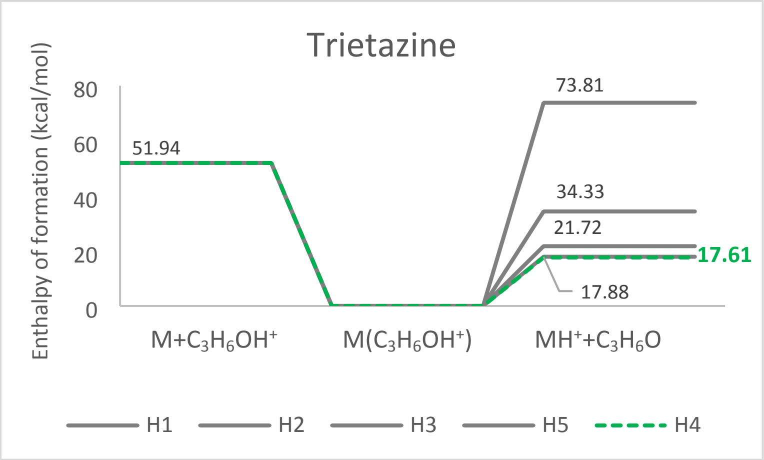

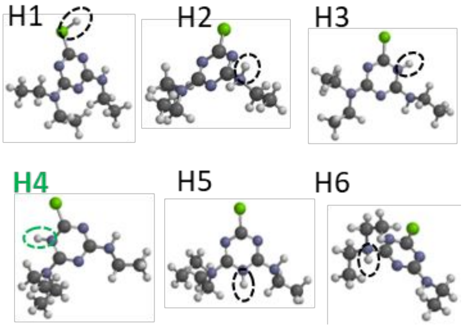

(b)

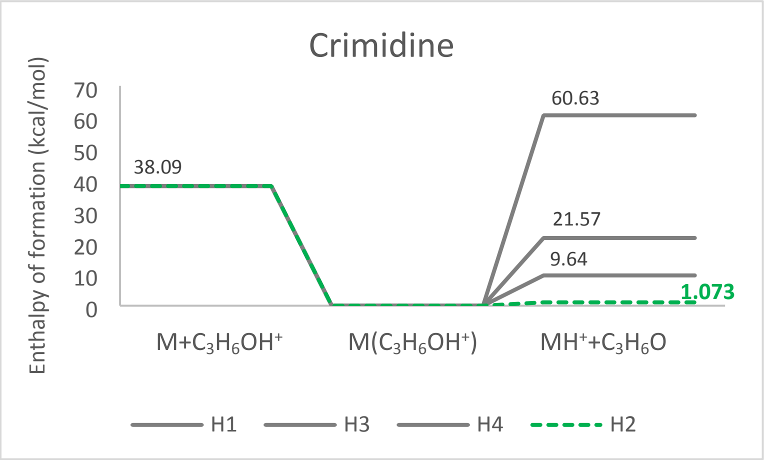

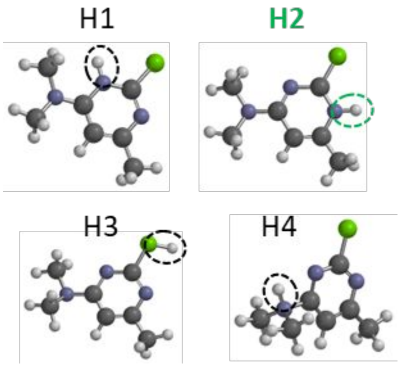

(c)

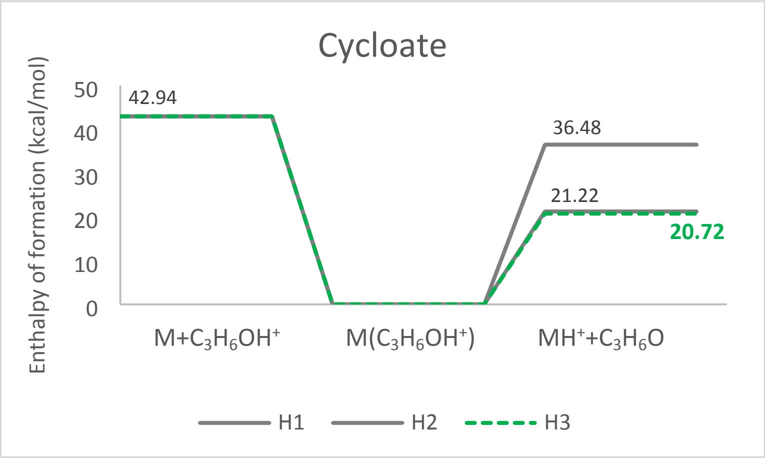

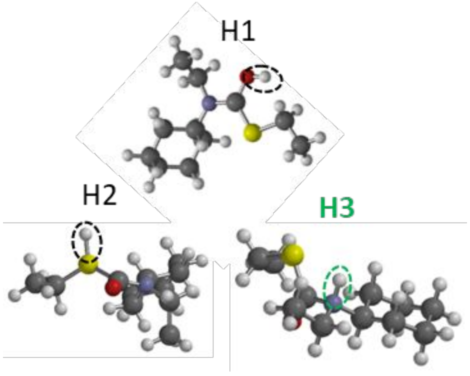

(d)

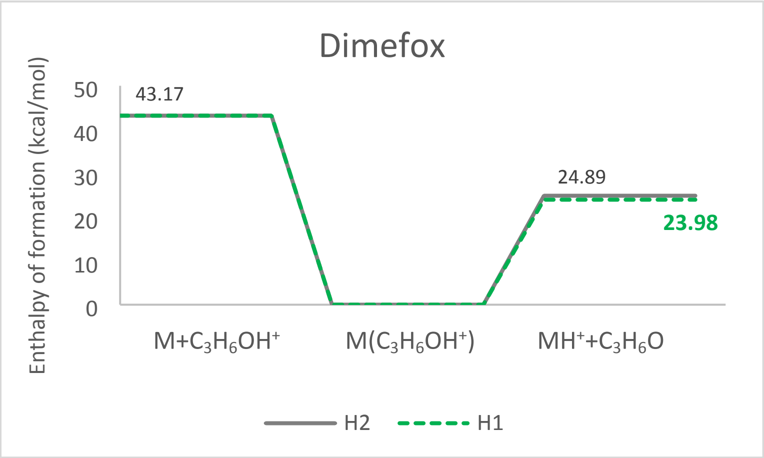

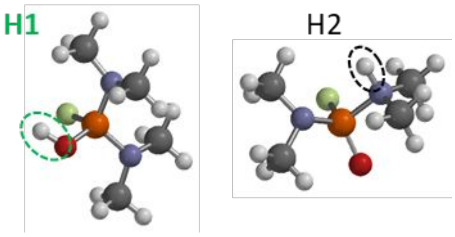

Table S5. Proton affinities of crimidine, trietazine, cycloate, dimefox, and acetone. All 4 pesticides have higher proton affinity than that of acetone, supporting preferential formation of protonated pesticide upon fragmentation.

Name	Proton affinity (kcal/mol)
Acetone	194.83
Dimefox	212.95
Cycloate	215.98
Trietazine	228.087
Crimidine	230.78

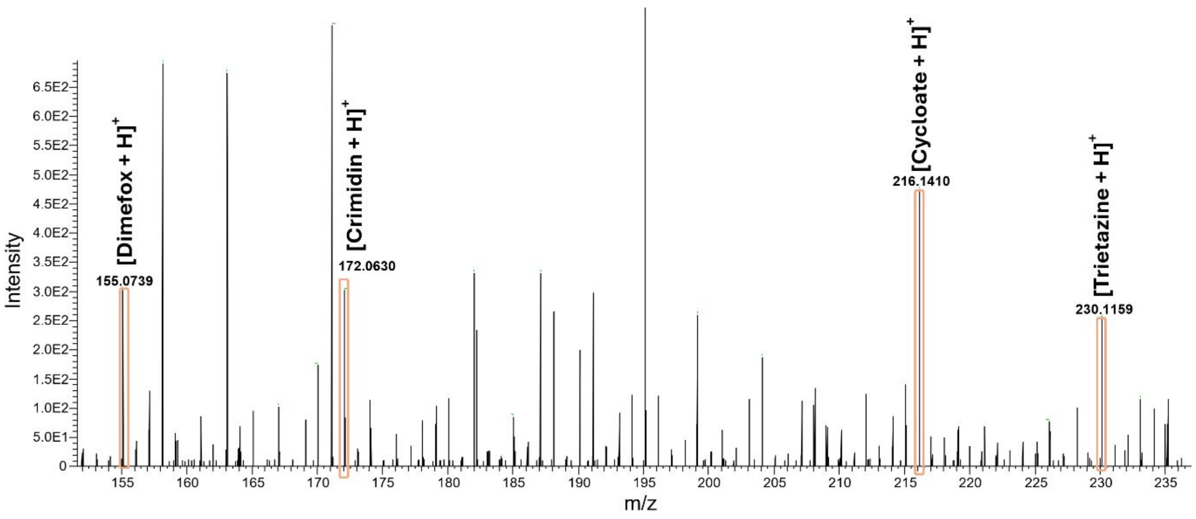

Figure S3. Protonated acetone chemical ionization mass spectrum showing the detection of of crimidine, trietazine, cycloate, and dimefox, displaying only the protonated pesticide peaks, as adducts with protonated acetone were not detected.

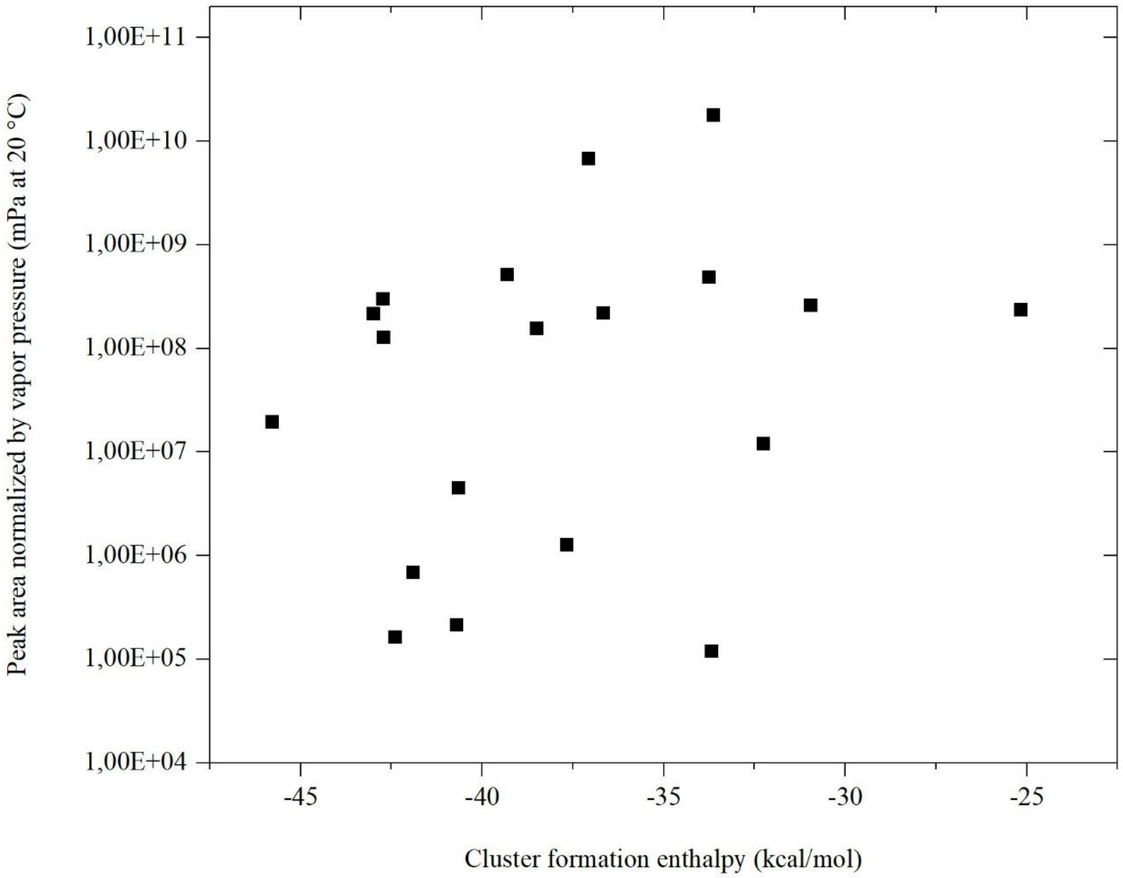

Figure S4. Calculated cluster formation enthalpies against experimental detection sensitivity (integrated peak area, PA, normalized by vapor pressure) for pesticides forming Br^{-} adducts. A linear correlation was not observed in these results.

Table S5. Values of cluster formation enthalpies (kcal/mol), vapor pressure at 20 °C (mPa), peak area under the desorption profile, and peak area normalized by vapor pressure.

Name	Cluster formation enthalpy (kcal/mol)	Peak area normalized by vapor pressure	Vapor pressure (mPa @ 20 °C)	Peak area
Fluometuron	-33.61	1.78E+10	1.00E-06	17800
Propanil	-37.05	6.78E+09	2.70E-06	18300
Fenpiclonil	-33.74	4.80E+08	4.60E-05	22100
TFNG	-39.30	5.11E+08	4.60E-05	23500
Sulfoxaflor	-42.97	2.15E+08	1.10E-04	23600
Flonicamid	-36.64	2.18E+08	1.20E-04	26100
Terbacil	-30.93	2.59E+08	1.30E-04	33700
Fipronil_sulfon	-25.15	2.35E+08	1.56E-04	36600
Fipronil_sulfide	-42.71	3.00E+08	2.00E-04	59900
Fluxapyroxad	-38.49	1.55E+08	3.90E-04	60300
Fipronil_desulfinyl	-42.69	1.28E+08	5.30E-04	67700
Chloridazon	-32.24	1.19E+07	9.10E-04	10800
Forchlorfenuron	-45.76	1.94E+07	1.40E-03	27200
Flupyradifurone	-40.63	4.47E+06	2.55E-03	11400
Triflumuron	-37.66	1.26E+06	1.33E-02	16700
Bixafen	-41.89	6.86E+05	5.10E-02	35000
Tebufenozide	-40.70	2.11E+05	1.25E-01	26400
Methoxyfenozide	-42.38	1.62E+05	1.30E-01	21000
Butoxycarboxim	-33.67	1.19E+05	1.93E-01	22900
